# DDX59-AS1 is a prognostic biomarker and correlated with immune infiltrates in OSCC

**DOI:** 10.3389/fgene.2022.892727

**Published:** 2022-08-23

**Authors:** Yang Sun, Qianrong Zhou, Jian Sun, Wei Bi, Ruixue Li, Xingwen Wu, Ni Li, Liang Song, Fei Yang, Youcheng Yu

**Affiliations:** Department of Stomatology, Zhongshan Hospital, Fudan University, Shanghai, China

**Keywords:** OSCC, TCGA, biomarker, immune infiltrates, DDX59-AS1

## Abstract

**Background:** lncRNAs play a critical role in multiple steps of gene regulation associated with tumor progression. However, the engagement of DDX59-AS1, a lncRNA, remains equivocal, particularly in oral squamous cell carcinoma (OSCC). In this study, the expression of DDX59-AS1 and its association with immune infiltration were investigated, and its prognostic value in OSSC was evaluated.

**Methods:** OSCC patients were collected from The Cancer Genome Atlas (TCGA) database. The expression of DDX59-AS1 in OSCC and healthy tissue was compared using Wilcoxon rank sum test. The relationship between DDX59-AS1 and clinicopathological features was analyzed using Logistic regression. Gene ontology (GO) terminology analysis, gene set enrichment analysis (GSEA), and single sample GSEA (ssGSEA) were utilized to interpret the enrichment pathway and functionality and to quantify the immune cell infiltration of DDX59-AS1. The correlation between survival and DDA59-AS1 was evaluated by Kaplan-Meier analysis and Cox regression. The prognostic impact of DDX59-AS1 was predicted by the nomogram based on Cox multivariate analysis.

**Results:** High expression of DDX59-AS1 was significantly correlated with T stage, clinical stage, race, and age (*p* < 0.05). Multivariate survival analysis demonstrated that the high expression of DDX59-AS1 was associated with lower overall and specific survival rates. The prognosis prediction was validated by the nomogram and calibration curves. The expression of DDX59-AS1 was negatively correlated with Mast cells, Tfh, T cells, Treg, and B cells, and positively related with the Tgd infiltration level.

**Conclusion:** DDX59-AS1 played a crucial role in the progression and prognosis of OSCC and was potentially a predictive biomarker for OSCC.

## Introduction

Oral squamous cell carcinoma (OSCC) is a prevalent oral malignancy worldwide, with 500,000 new cases and 140,000 deaths occurring annually (Zhang et al., 2021). Despite the prognosis could be improved *via* multidisciplinary treatment and sequential therapy, the five-year survival rate remains at 50%, and local recurrence and distant metastasis develop in 25%–50% of patients (Ma et al., 2020). While early diagnosis has resulted in a significant positive impact on the prognosis, OSCC patients are typically presented at a late stage due to the asymptomatic nature of the disease and their lack of awareness of the disease’s risk factors, leading to a poor prognosis and increased mortality (Manzano-Moreno et al., 2021), with only a small portion of the OSCC patients seeing positive outcomes from the treatment. Therefore, it is important to identify the predictive biomarker and potential therapeutic targets of the disease.

In addition to the studies on OSCC concentrated on the protein-coding genes, it has been revealed that the non-coding RNAs (ncRNAs), especially long-stranded non-coding RNAs (lncRNAs) played a crucial role in the prognosis of many malignancies (Liang et al., 2020). LncRNAs are involved in different types of biological processes, including cellular proliferation and apoptosis, growth and development of the body, inflammatory response, and the occurrence and development of cancer and other diseases. In fact, lncRNA has been functionally implicated as a regulator of the tumorigenesis and development; therefore, it has been considered as a novel target for the development of anti-cancer therapeutics. However, the role of lncRNA in OSCC prognosis remains to be explored. Therefore, investigating the expression of lncRNAs in OSCC can potentially provide critical experimental data and a theoretical basis for the prevention and treatment of OSCC.

DDX59 antisense RNA (DDX59-AS1) is located in 32.1 regions of the long arm of chromosome 1, which is strongly related to nicotine dependency, as described by GeneCards. While smoking is a crucial element in the triggering of OSCC, the role of DDX59-AS1 in this process has not been reported. Therefore, it is critical to elucidate the involvement of DDX59-AS1 in malignancies, particularly in OSCC.

**FIGURE 1:**
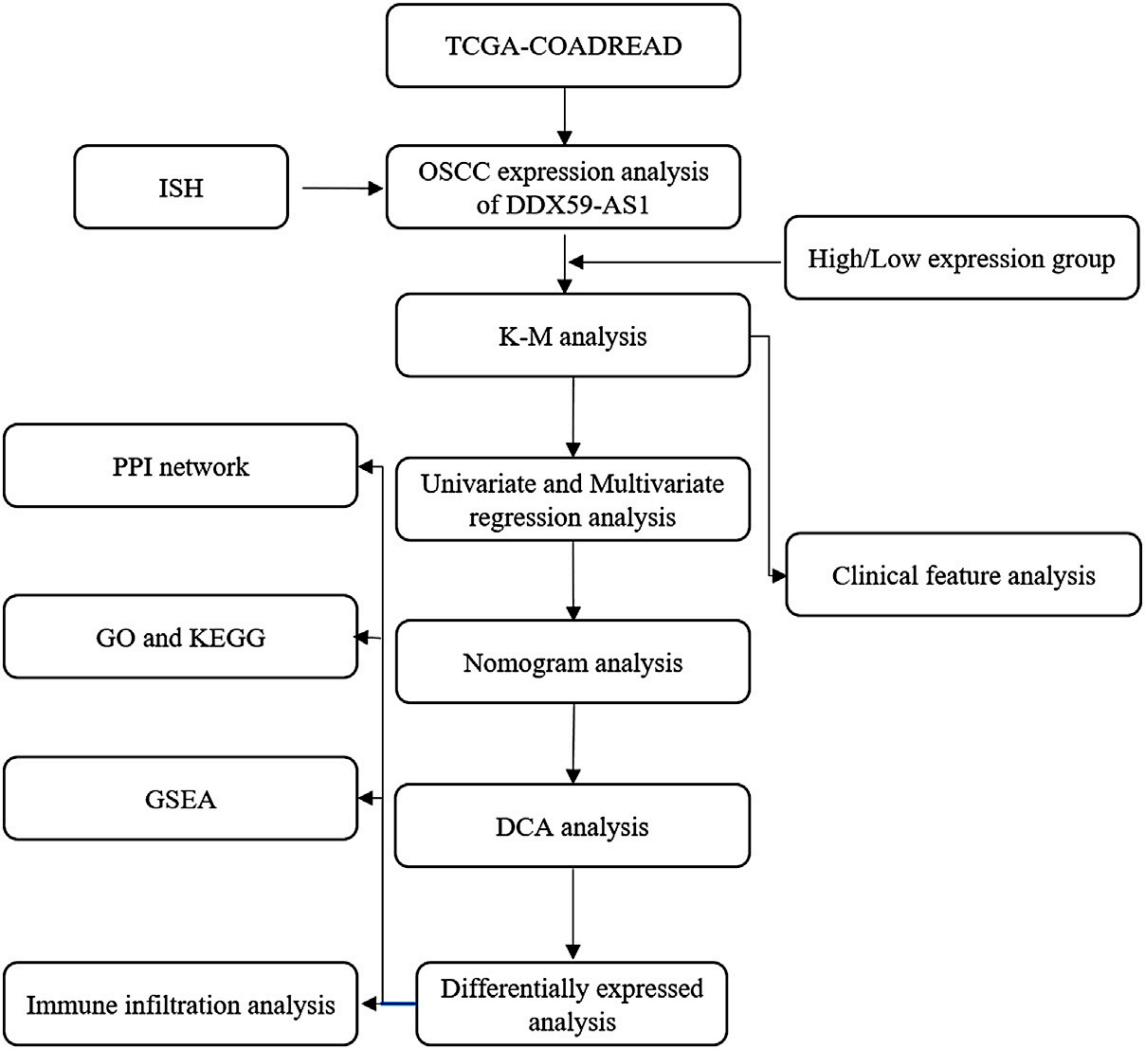
Analysis flow chart.

In this study, the clinical correlation between the expression levels of DDX59-AS1 and OSCC was explored using The Cancer Genome Atlas (TCGA) database. It was identified that the increased expression of DDX59-AS1 was positively correlated to the prognosis of overall survival (OS) and disease-specific survival (DSS). In addition, the difference between healthy and OSCC tissue in DDX59-AS1 was analyzed. Gene and function set enrichment analysis was performed on the groups distinguished by high or low expression of DDX59-AS1 to reveal its potential functionality. Finally, the correlation between DDX50-AS1 expression and OSCC immune infiltration was analyzed, and the mechanism of DDX59-AS1 in OSCC was analyzed collectively. In summary, this study identified DDX59-AS1 as a significant independent predictor of OSCC, and the inhibition of DDX59-AS1 is a potential target for the treatment of OSCC. The flow chart of our work was shown in Figure 1.

## Material and methods

### Data source and preprocessing

RNAseq data in level 3 HTSeq-FPKM (Fragments Per Kilobase per Million) format and clinical information were downloaded from the Head and Neck Squamous Cell Carcinoma (HNSC) project of TCGA (https://portal.gdc.cancer.gov/). A total of 331 samples with clinical information were proceeded for analysis, including the oral cavity (alveolar ridge, base of tongue, buccal mucosa, floor of mouth, hard palate, lip, oral cavity, and oral tongue) samples, and excluding the non-oral (Hypopharynx, Larynx, Oropharynx, Tonsil) samples. The data was converted to TPM (transcripts per million reads) format for inter-sample comparison on expression. Gene expression data were divided into a high and low expression group according to the median DDX59-AS1 expression level. This study met the publication guidelines stated by TCGA (https://cancergenome.nih.gov/publications/publication guidelines). All data used were acquired from TCGA; hence, ethical approval and informed consent of the patients were not required.

### Differentially expressed genes analysis

The expression profile between high/low DDX59-AS1 expression groups was compared *via* HTSeq-Counts to identify the differentially expressed genes (DEG) using Wilcoxon rank sum test in DESeq2 (3.8) based on R. |logFC|>1 and adjusted *p* < 0.05 were considered as the threshold to identify DEGs. Clusterprofile 3.6.0 was utilized to perform gene ontology (GO) functional enrichment analysis and Kyoto Encyclopedia of Genes and Genomes (KEGG) analysis on the DEGs identified in the high/low DDX59-AS1 expression groups. STRING database 10.0 (http://string-db.org) for protein-protein interaction (PPI) networks functional enrichment analysis was utilized to predict the PPI network of the co-expressed genes of DDX59-AS1 and to analyze the functional interactions between proteins. Interactions with a threshold >0.4 were considered statistically significant.

### *In situ* hybridization analysis

Paired tumor and adjacent tissue samples were collected from 20 patients diagnosed with OSCC. All samples were obtained from the Department of Stomatology, Zhongshan Hospital Affiliated to Fudan University, and were approved by the Ethics Committee. The probe sequence of DDX59-AS1 is：5′-CTTACTGGATCTTGTGCCTAAGAAGCCCAA-3′. Staining results were evaluated by two independent pathologists. Staining intensity (0, no staining; 1+, weak staining; 2+, moderate staining; 3+, strong staining) and percentage of cells stained (0, <5%; 1, 5%–25%; 2, 26%–50%; 3, 51%–75%; and 4, >75%). The staining intensity score was multiplied by the percentage of positive cells to generate a score for each sample.

### Gene set enrichment analysis

GSEA is a computational method to determine whether a defined set of genes show statistically significant and consistent differences between two biological states. In this study, GSEA was used to elucidate the significant functional and pathway differences between high and low DDX59-AS1 expression groups *via* a DDX59-AS1 differential expression matrix. Each analysis procedure was repeated 1,000 times. Enrichment of function or pathway terms with an adjusted *p* < 0.05 and FDR<0.25 were considered statistically significant.

### Immune infiltration analysis

Tumor infiltration levels of 24 immune cell types were quantified by ssGSEA. Expression levels of the characteristic genes were acquired in published literature. The correlation between DDX59-AS1 and the 24 types of cells was analyzed by Spearman correlation method. The level of immune cell infiltration was calculated using the CIBERSORT algorithm (Chen et al., 2018). Differences in infiltration levels were calculated by Wilcoxon test. The Spearman correlation method was used to analyze the correlation between DDX59-AS1 and immune cells. In addition, we used Estimate to calculate the immune and stromal scores of the specimens by RNA-seq to assess the purity of the tumor. We used ImmuneScore, StromalScore, ESTIMA Score, and tumor purity to quantify the immune and stromal components of tumor species (Yoshihara et al., 2013).

### Building prognostic model and statistical analysis of relevant clinical data

The relationship between DDX59-AS1 and clinicopathological characteristics of OSCC was analyzed using Wilcoxon signed-rank test and Logistic regression. OS and DSS related clinicopathological characteristics were analyzed using Cox regression and Kaplan-Meier methods. Multivariate Cox analysis was used to compare the impact of DDX59-AS1 expression on survival and other clinical characteristics. In all the analyses above, *p* < 0.05 was considered significant.

Independent prognostic factors obtained from multivariate analyses based on Cox regression models were used to create a nomogram and to individualize the predicted survival probabilities at 1, 3, and 5 years. The nomogram with critical clinical features and calibration curves were generated using the RMS package. The calibration curve evaluates the prediction by mapping the predicted probability from the nomogram to the observed events, a 45° line indicate the best predicted value. The discriminatory power of the line plot was determined using consistency index (C-index), which was calculated by the bootstrap method with 1,000 resamples. Finally, the accuracy of the prediction of nomogram and the individual prognostic factors was compared using the C-index. In this study, all the statistical tests were two-tailed, and the level of statistical significance was set at 0.05.

## Results

### DDX59-AS1 showed significantly high expression in OSCC samples

**FIGURE 2:**
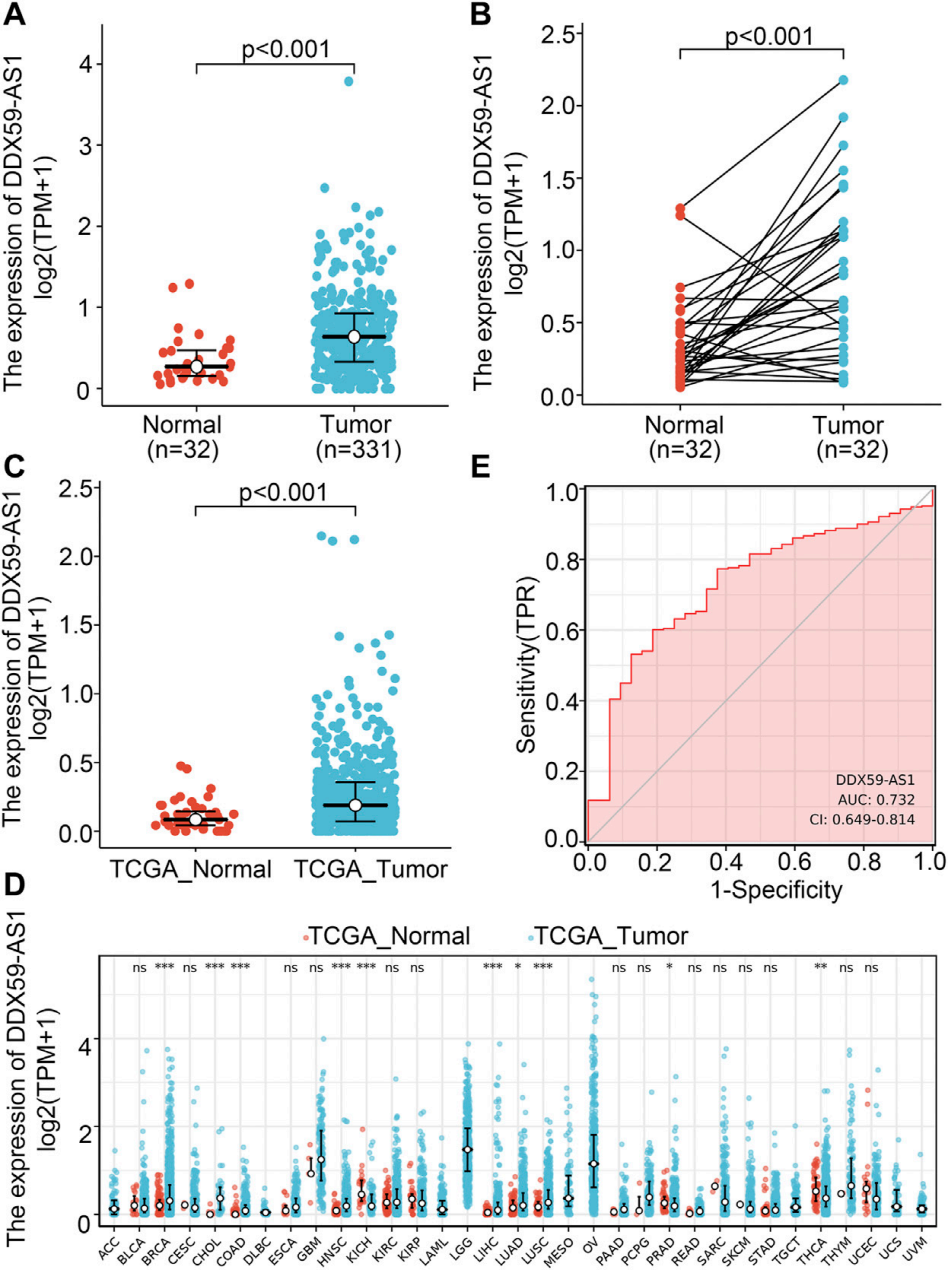
DDX59-AS1 expression between cancer and normal tissues in OSCC patients. **(A)** DDX59-AS1 expression levels in OSCC and normal tissues; **(B)** DDX59-AS1 expression levels in OSCC and matched normal tissues; **(C)** DDX59-AS1 expression between cancer and normal tissues in HNSCC; **(D)** The comparison of DDX59-AS1 expression between cancer and normal tissue in different types of cancers based on TCGA database; **(E)** ROC analysis of DDX59-AS1 shows promising discrimination power between cancer and normal tissues. ns, *p* ≥ 0.05; *, *p* < 0.05; **, *p* < 0.01; ***, *p* < 0.001.

With its unknown expression and functionality in malignancy, DDX59-AS1 was proposed as the target gene in this study for any potential effect in the diagnosis and prognosis of OSCC. Wilcoxon rank sum test was utilized to determine the difference of DDX59-AS1 expression between normal and cancerous tissue among the 32 paraneoplastic samples and 331 OSCC samples analyzed (Yin et al., 2021). DDX59-AS1 showed significantly increased expression in the OSCC samples (*p* < 0.001, Figure 2A). The increase was also observed in the 32 OSCC samples with paired paraneoplastic samples (*p* < 0.001, Figure 2B). The expression of DDX59-AS1 was also compared in head and neck squamous carcinoma tissue in head and neck squamous cell carcinoma (HNSCC) (*p* < 0.001, Figure 2C). In addition, by comparing the DDX59-AS1 transcription level in the malignancy tissue to that in the normal tissue in the TCGA database, we found that DDX59-AS1 showed a high expression in a variety of types of cancers, including breast invasive carcinoma (BRCA), cholangiocarcinoma (CHOL), colon adenocarcinoma (COAD), head and neck squamous cell carcinoma (HNSC), kidney chromophobe (KICH), liver hepatocellular carcinoma (LIHC), lung adenocarcinoma (LUAD), lung squamous cell carcinoma (LUSC), prostate adenocarcinoma (PRAD), and thyroid cancer (THCA) (*p* < 0.05, Figure 2D).

**FIGURE 3:**
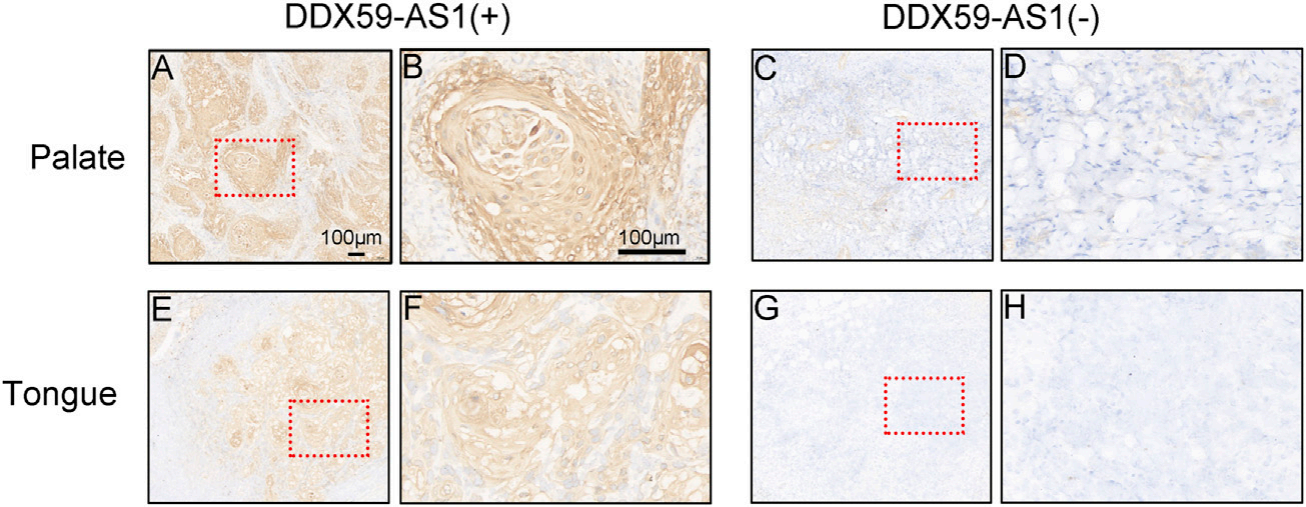
Expression of DDX59-AS1 in OSCC and adjacent tissues. **(A–B)** Expression of DDX59-AS1 in palate carcinoma; **(C–D)** Expression of DDX59-AS1 in the adjacent palatal tissue; **(E–F)** Expression of DDX59-AS1 in tongue cancer tissue; **(G–H)** Expression of DDX59-AS1 in tongue adjacent tissue.

Receiver operating characteristic (ROC) curve identified the expression level of DDX59-AS1 as a potential diagnosis molecule to distinguish between malignancy and normal tissue, with the area under the curve (AUC) of 0.732, which indicated high specificity and sensitivity (Figure 2E). To further determine the importance of DDX59-AS1 expression, *in situ* hybridization (ISH) staining was performed on cancer tissues from 20 OSCC patients with paired adjacent tissues. According to the staining results, DDX59-AS1 was positive in the cytoplasm of cancer tissues but negative in adjacent tissues. A Representative image was shown in Figure 3.

### DDX59-AS1 expression correlated with unfavorable clinicopathological features of OSCC

**FIGURE 4:**
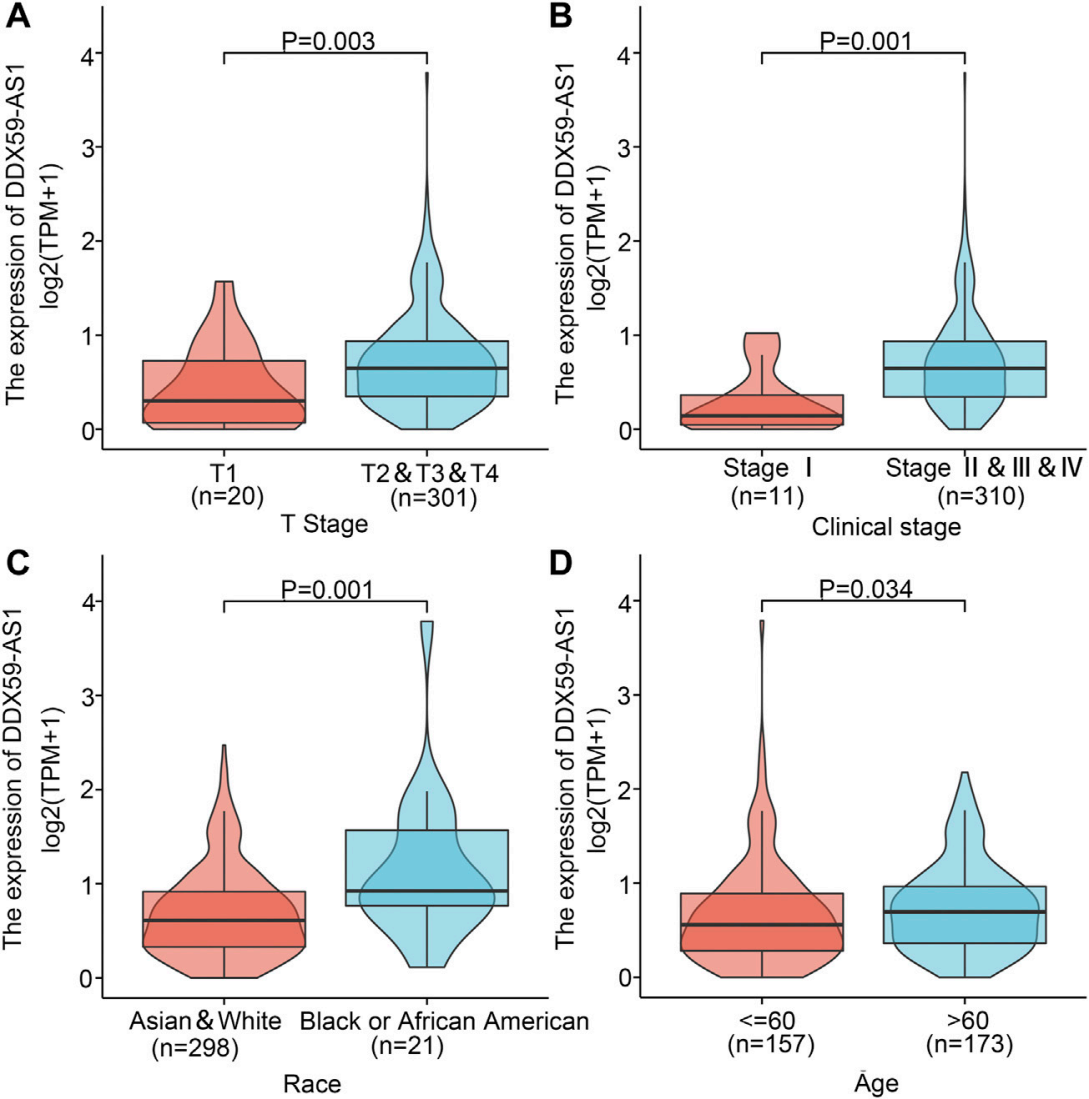
Association of DDX59-AS1 expression with clinicopathologic characteristics. **(A)** T stage; **(B)** Clinical stage; **(C)** Race; **(D)** Age.

The patients were divided into high (*n* = 165) and low (*n* = 166) DDX-AS1 expression groups based on their mean DDX59-AS1 relative expression. The clinicopathological characteristics of the two groups were investigated respectively, as shown in Table 1. Chi-square test and Fisher’s exact test demonstrated that the expression of DDX59-AS1 was correlated with race (*p* = 0.001) and anatomic neoplasm subdivision (*p* < 0.001). In addition, the DDX59-AS1 expression in patients with different clinicopathological characteristics was analyzed. As validated by Wilcoxon rank sum test, the expression of DDX59-AS1 was significantly increased in T2, T3, and T4 of T stage (*p* = 0.003), Clinical stage II, III, and IV (*p* = 0.001), black or African American (*p* = 0.001) and age ≤60 years (*p* = 0.034) (Figure 4). The correlation between clinical pathological features of OSCC and the expression level of DDX59-AS1 was analyzed by Logistics regression, verifying a significant correlation of DDX59-AS1 expression with the clinical stage (*p* = 0.047), primary therapy outcome (*p* = 0.044), and a moderate correlation with T stage (*p* = 0.066) (Table 2).

**TABLE 1:** Clinicopathological characteristics of OSCC patients with differential DDX59-AS1 expression.

Characters	Level	Low expression of DDX59-AS1	High expression of DDX59-AS1	*p*	Test
*n*		166	165		
T stage (%)	T1	14 (8.8%)	6 (3.7%)	0.308	
	T2	50 (31.4%)	55 (34.0%)		
	T3	40 (25.2%)	42 (25.9%)		
	T4	55 (34.6%)	59 (36.4%)		
N stage (%)	N0	81 (51.3%)	87 (54.7%)	0.868	Exact
	N1	29 (18.4%)	29 (18.2%)		
	N2	46 (29.1%)	42 (26.4%)		
	N3	2 (1.3%)	1 (0.6%)		
M stage (%)	M0	157 (100.0%)	155 (98.7%)	0.498	Exact
	M1	0 (0.0%)	2 (1.3%)		
Clinical stage (%)	Stage I	9 (5.7%)	2 (1.2%)	0.125	Exact
	Stage II	35 (22.0%)	44 (27.2%)		
	Stage III	32 (20.1%)	36 (22.2%)		
	Stage IV	83 (52.2%)	80 (49.4%)		
Primary therapy outcome (%)	CR	124 (89.2%)	114 (80.9%)	0.210	Exact
	PD	12 (8.6%)	23 (16.3%)		
	PR	1 (0.7%)	2 (1.4%)		
	SD	2 (1.4%)	2 (1.4%)		
Gender (%)	Female	53 (31.9%)	50 (30.3%)	0.841	
	Male	113 (68.1%)	115 (69.7%)		
Race (%)	Asian	6 (3.8%)	3 (1.9%)	0.001	Exact
	Black or African American	3 (1.9%)	18 (11.2%)		
	White	149 (94.3%)	140 (87.0%)		
Age (%)	≤60	87 (52.4%)	70 (42.7%)	0.097	
	>60	79 (47.6%)	94 (57.3%)		
Histologic grade (%)	G1	24 (14.8%)	27 (16.8%)	0.557	Exact
	G2	104 (64.2%)	98 (60.9%)		
	G3	32 (19.8%)	36 (22.4%)		
	G4	2 (1.2%)	0 (0.0%)		
Anatomic neoplasm subdivision (%)	Alveolar ridge	8 (4.8%)	10 (6.1%)	NA	Exact
	Base of tongue	17 (10.2%)	6 (3.6%)		
	Buccal mucosa	11 (6.6%)	11 (6.7%)		
	Floor of mouth	24 (14.5%)	36 (21.8%)		
	Hard palate	5 (3.0%)	2 (1.2%)		
	Lip	3 (1.8%)	0 (0.0%)		
	Oral cavity	30 (18.1%)	42 (25.5%)		
	Oral tongue	68 (41.0%)	58 (35.2%)		
Smoker (%)	No	47 (28.5%)	40 (25.0%)	0.559	
	Yes	118 (71.5%)	120 (75.0%)		
Alcohol history (%)	No	56 (33.9%)	50 (31.6%)	0.749	
	Yes	109 (66.1%)	108 (68.4%)		
Perineural invasion (%)	No	61 (50.0%)	56 (43.4%)	0.358	
	Yes	61 (50.0%)	73 (56.6%)		
Lymphovascular invasion (%)	No	79 (66.4%)	86 (71.7%)	0.458	
	Yes	40 (33.6%)	34 (28.3%)		
TP53 status (%)	Mut	111 (67.3%)	112 (69.1%)	0.808	
	WT	54 (32.7%)	50 (30.9%)		
PIK3CA status (%)	Mut	29 (17.6%)	24 (14.8%)	0.598	
	WT	136 (82.4%)	138 (85.2%)		
Age (median [IQR])		60.00 [53.00,69.00]	62.00 [55.00,72.25]	0.111	Non-norm

IQR, interquartile range.

**TABLE 2:** Logistic regression analysis of association between clinicopathological characteristics and DDX59-AS1 expression in OSCC patients.

Characteristics	Odds ratio in DDX59-AS1 expression	Odds ratio (OR)	*p* value
T stage (T2&T3&T4 vs. T1)	321	2.51 (0.98–7.25)	0.066
N stage (N1&N2&N3 vs. N0)	317	0.87 (0.56–1.35)	0.538
M stage (M1 vs. M0)	314	81473679.90 (0.00-NA)	0.996
Clinical stage (Stage II&Stage III&Stage IV vs. Stage I)	321	4.80 (1.21–31.82)	0.047
Primary therapy outcome (CR vs. PD&PR)	276	0.48 (0.23–0.96)	0.044
Histologic grade (G3&G4 vs. G1&G2)	323	1.08 (0.64–1.85)	0.765
Perineural invasion (Yes vs. No)	251	1.30 (0.79–2.15)	0.296
Lymphovascular invasion (Yes vs. No)	239	0.78 (0.45–1.35)	0.378
TP53 status (Mut vs. WT)	327	1.09 (0.68–1.74)	0.718
PIK3CA status (Mut vs. WT)	327	0.82 (0.45–1.47)	0.499

WT, wild type; Mut, mutation.

### High expression of DDX59-AS1 predicted poor prognosis of OSCC

**FIGURE 5:**
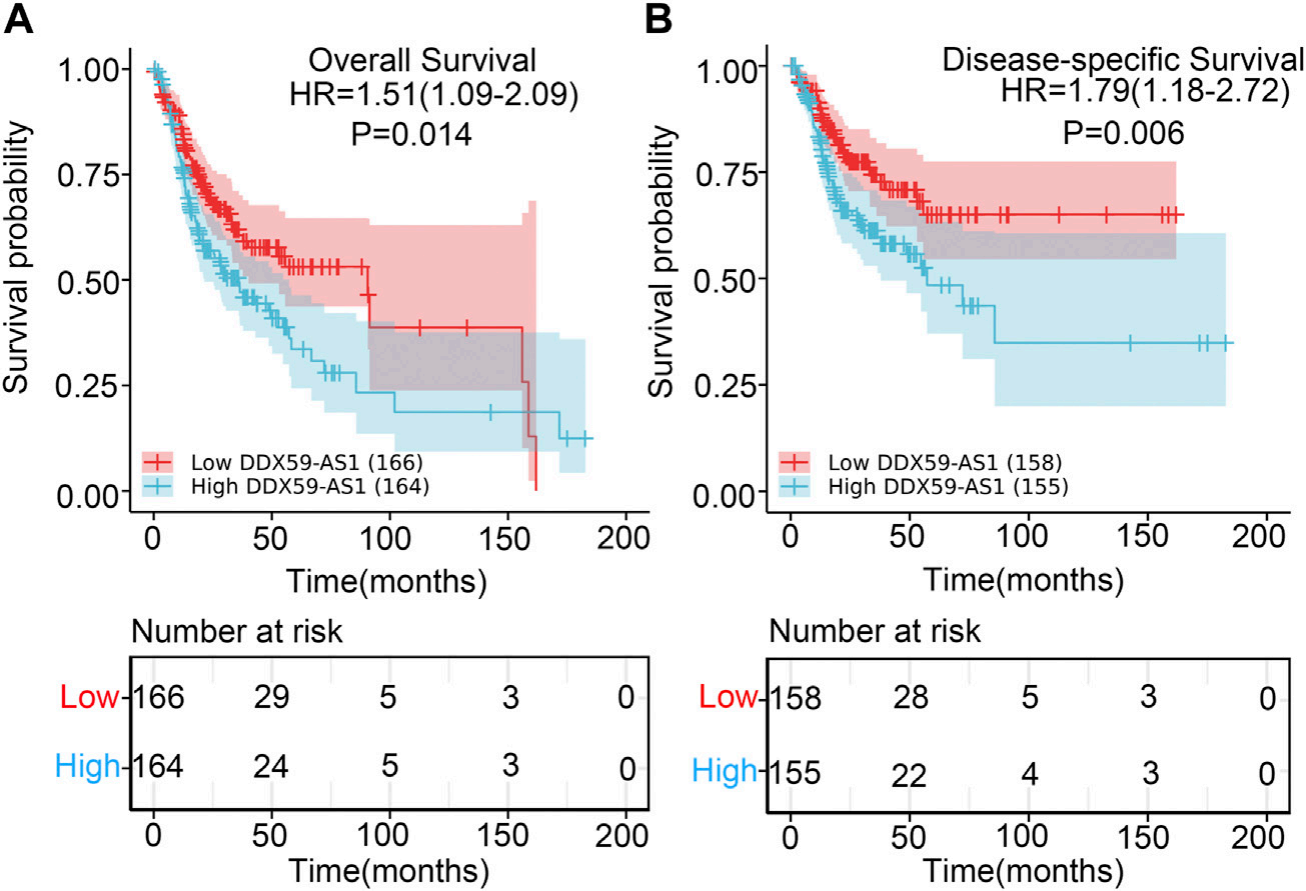
Kaplan-Meier survival curves comparing the high and low expression of DDX59-AS1 in OSCC patients. **(A)** Overall survival; **(B)** Disease-Specific Survival.

The prognostic value of DDX59-AS1 in the overall and disease-specific survival was evaluated with Kaplan-Meier plotting (Chen et al., 2021). As shown in Figures 5A,B, the high expression of DDX59-AS1 was correlated with unfavorable OS (HR = 1.51, *p* = 0.014) and DSS (HR = 1.79, *p* = 0.006). To further assess the predictive value of DDX59-AS1, variables with *p* < 0.05 in the univariate cox regression, including primary therapy outcome (*p* < 0.001), perineural invasion (*p* = 0.002), lymphovasuclar invasion (*p* = 0.012), and DDX-AS1 (*p* = 0.014), were proceeded for multivariate cox regeression (Ai et al., 2021). Collectively, it was indicated that primary therapy outcome (*p* < 0.001), perineural invasion (*p* = 0.018) and DDX-AS1 (*p* = 0.013) were independent prognostic factor in overall survival (*p* < 0.05) (Table 3). The independent prognostic factors of DDS were listed in Supplementary Table S1.

**TABLE 3:** Cox regression analysis for clinical outcomes in OSCC patients.

Characteristics	Total (N)	HR (95%CI) univariate analysis	*p* Value univariate analysis	HR (95%CI) multivariate analysis	*p* value multivariate analysis
T stage (T3&T4 vs. T1&T2)	320	1.379 (0.983–1.937)	0.063	1.334 (0.822–2.167)	0.243
N stage (N1&N2&N3 vs. N0)	316	1.297 (0.939–1.793)	0.115		
M stage (M1 vs. M0)	313	2.662 (0.370–19.131)	0.331		
Clinical stage (Stage III&Stage IV vs. Stage I&Stage II)	320	1.269 (0.880–1.829)	0.202		
Primary therapy outcome (CR vs. PD&SD&PR)	279	0.168 (0.109–0.259)	<0.001	0.222 (0.134–0.366)	<0.001
Histologic grade (G3&G4 vs. G1&G2)	322	1.246 (0.857–1.811)	0.249		
Gender (Male vs. Female)	330	0.909 (0.648–1.273)	0.578		
Race (White vs. Asian&Black or African American)	319	0.641 (0.368–1.116)	0.116		
Age (>60 vs.≤60)	330	1.290 (0.933–1.784)	0.123		
Smoker (Yes vs. No)	324	1.235 (0.836–1.824)	0.288		
Alcohol history (Yes vs. No)	322	1.093 (0.773–1.546)	0.615		
Perineural invasion (Yes vs. No)	250	1.924 (1.282–2.886)	0.002	1.812 (1.107–2.966)	0.018
Lymphovascular invasion (Yes vs. No)	238	1.671 (1.119–2.496)	0.012	1.441 (0.915–2.270)	0.115
TP53 status (Mut vs. WT)	327	1.271 (0.881–1.835)	0.200		
PIK3CA status (Mut vs. WT)	327	1.061 (0.703–1.602)	0.777		
DDX59-AS1 (High vs. Low)	330	1.507 (1.088–2.087)	0.014	1.776 (1.129–2.791)	0.013

HR, hazard ratio; WT, wild type; Mut, mutation; CI, confidence interval.

### Prognosis performance of DDX59-AS1 in the OSCC clinicopathological subgroup

**FIGURE 6:**
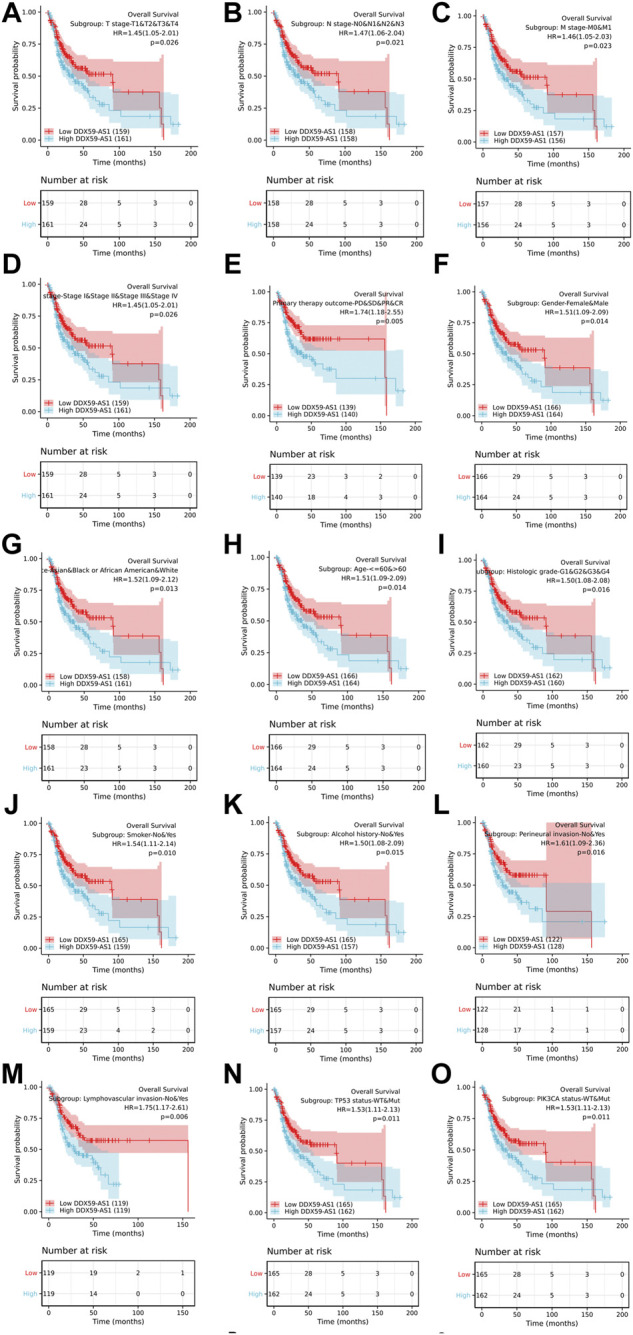
Overall survival for subgroup analyses. **(A)**T stage; **(B)** N stage; **(C)** M stage; **(D)** Clinical stage; **(E)** Primary therapy outcome; **(F)** Gender; **(G)** Race; **(H)** Age; **(I)** Histologic grade; **(J)** Smoker; **(K)** Alcohol; **(L)** Perineural invasion; **(M)** Lymph vascular invasion; **(N)** TP53 status; **(O)** PIK3CA status.

Kaplan-Meier analysis was utilized to evaluate the predictive value of DDX59-AS1 for overall and specific survival of clinical outcomes in several clinicopathological subgroups (Figure 6, Supplementary Figure S1). Cox regression was performed in specific subgroups. In terms of overall survival, DDX59-AS1 showed significance in the following subgroups: T2,3,4 stage [HR = 1.435 (1.026–2.006), *p* = 0.035], clinical stage IV [HR = 1.654 (1.057–2.589), *p* = 0.028], Male [HR = 1.526 (1.1019–2.285), *p* = 0.040], age, Yes (smoker) [HR = 1.507 (1.036–2.193), *p* = 0.032], alcohol history, No (lymphovascular invasion) [HR = 2.616 (1.523–4.494), *p* < 0.001], No (perineural invasion) [HR = 2.008 (1.010–3.992), *p* = 0.047], WT (TP53 status) [HR = 2.023 (1.037–3.946), *p* = 0.039], WT (PIK3CA status) [HR = 1.653 (1.146–2.383), *p* = 0.007], primary therapy outcome subgroups, G1&G2 (histologic grade) [HR = 1.615 (1.102–2.367), *p* = 0.014], white (race) [HR = 1.429 (1.011–2.019), *p* = 0.043], N2&N3 stage [HR = 1.889 (1.026–3.475), *p* = 0.041] (Table 4). The prognostic performance of DDX59-AS1 in disease-specific survival in the subgroups was listed in Supplementary Table S1.

**TABLE 4:** Overall survival prognostic performance of DDX59-AS1 on clinical outcomes in OSCC patient subgroups by Cox regression analysis.

Characteristics	N (%)	HR (95% CI)	*p* value
T stage			
T1	20 (6)	1.339 (0.258–6.939)	0.728
T2&T3&T4	300 (94)	1.435 (1.026–2.006)	0.035
Clinical stage			
Stage I	11 (3)	1.398 (0.154–12.656)	0.766
Stage II	78 (24)	1.413 (0.710–2.813)	0.325
Stage III	68 (21)	1.312 (0.631–2.730)	0.467
Stage IV	163 (51)	1.654 (1.057–2.589)	0.028
Gender			
Female	103 (31)	1.513 (0.869–2.634)	0.144
Male	227 (69)	1.526 (1.019–2.285)	0.040
Age			
≤60	157 (48)	1.417 (0.866–2.316)	0.165
>60	173 (52)	1.537 (0.990–2.387)	0.056
Smoker			
No	87 (27)	1.708 (0.848–3.443)	0.134
Yes	237 (73)	1.507 (1.036–2.193)	0.032
Alcohol history			
No	106 (33)	1.618 (0.909–2.881)	0.102
Yes	216 (67)	1.451 (0.972–2.165)	0.068
Lymphovascular invasion			
No	164 (69)	2.616 (1.523–4.494)	<0.001
Yes	74 (31)	0.955 (0.508–1.798)	0.887
Perineural invasion			
No	116 (46)	2.008 (1.010–3.992)	0.047
Yes	134 (54)	1.385 (0.870–2.204)	0.170
TP53 status			
WT	104 (32)	2.023 (1.037–3.946)	0.039
Mut	223 (68)	1.413 (0.965–2.069)	0.075
PIK3CA status			
WT	274 (84)	1.653 (1.146–2.383)	0.007
Mut	53 (16)	1.162 (0.553–2.443)	0.692
Primary therapy outcome			
PD&SD&PR	42 (15)	1.478 (0.677–3.225)	0.326
CR	237 (85)	1.491 (0.950–2.341)	0.083
Histologic grade			
G1&G2	252 (78)	1.615 (1.102–2.367)	0.014
G3&G4	70 (22)	1.191 (0.617–2.301)	0.602
Race			
Asian&Black or African American	30 (9)	2.418 (0.530–11.034)	0.254
White	289 (91)	1.429 (1.011–2.019)	0.043
N stage			
N0&N1	225 (71)	1.394 (0.942–2.064)	0.097
N2&N3	91 (29)	1.889 (1.026–3.475)	0.041

HR, hazard ratio; CI, confidence interval; WT, wild type; Mut, mutation.

### Establishment and validation of the nomogram based on DDX59-AS1 expression

**FIGURE 7:**
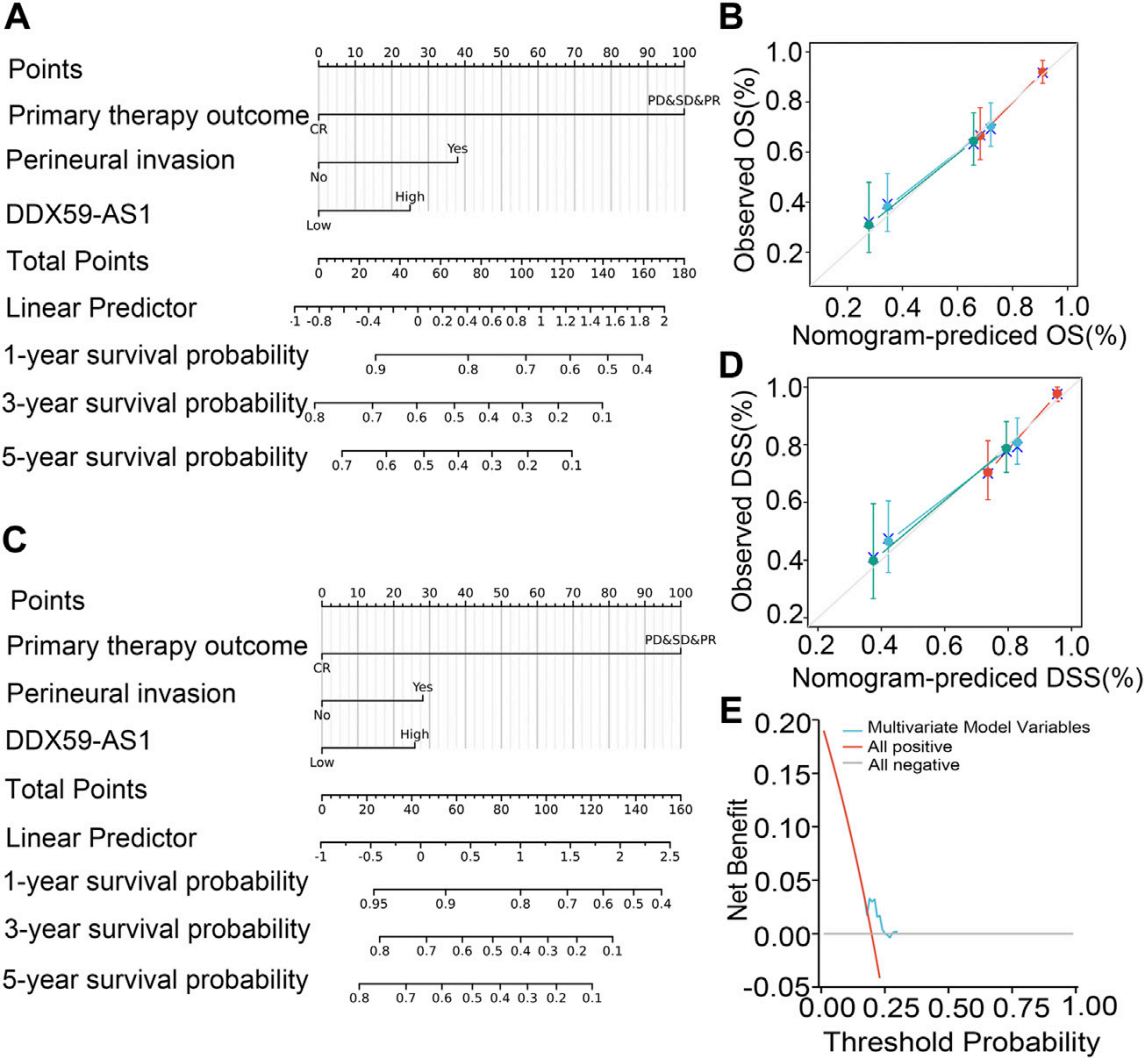
Relationship between DDX59-AS1 and other clinical factors with overall survival (OS). **(A)** Nomogram for predicting the probability of 1-,3-, and 5-year OS for OSCC patients; **(B)** Calibration plot of the nomogram for predicting the OS likelihood; **(C)** Nomogram for predicting the probability of 1-,3-, and 5-year DSS for OSCC patients; **(D)** Calibration plot of the nomogram for predicting the DSS likelihood; **(E)** DCA curve for evaluating nomogram.

The nomogram was established to predict the prognosis of OSCC patients and validated by its calibration curves. The nomogram integrated clinical characteristics independently associated with overall survival (C-index = 0.699) and disease-specific survival (C-index = 0.761) as identified by the multivariate analysis of primary therapy outcome, perineural invasion, and DDX59-AS1. The calibration curve of the nomogram of 1-, 3-, 5- year clinical outcomes all indicated a good agreement between the predicted and observed values, with the deviation-corrected lines approaching the 45-degree line. Consistent with this result, the DCA figure also proved that the nomogram combined with various clinical features has better clinical application value. Overall, the nomogram was an advanced model for predicting long-term survival in OSCC patients compared to individual prognostic factors (Figure 7).

### DEG identification between high/low DDX59-AS1 expression groups

**FIGURE 8:**
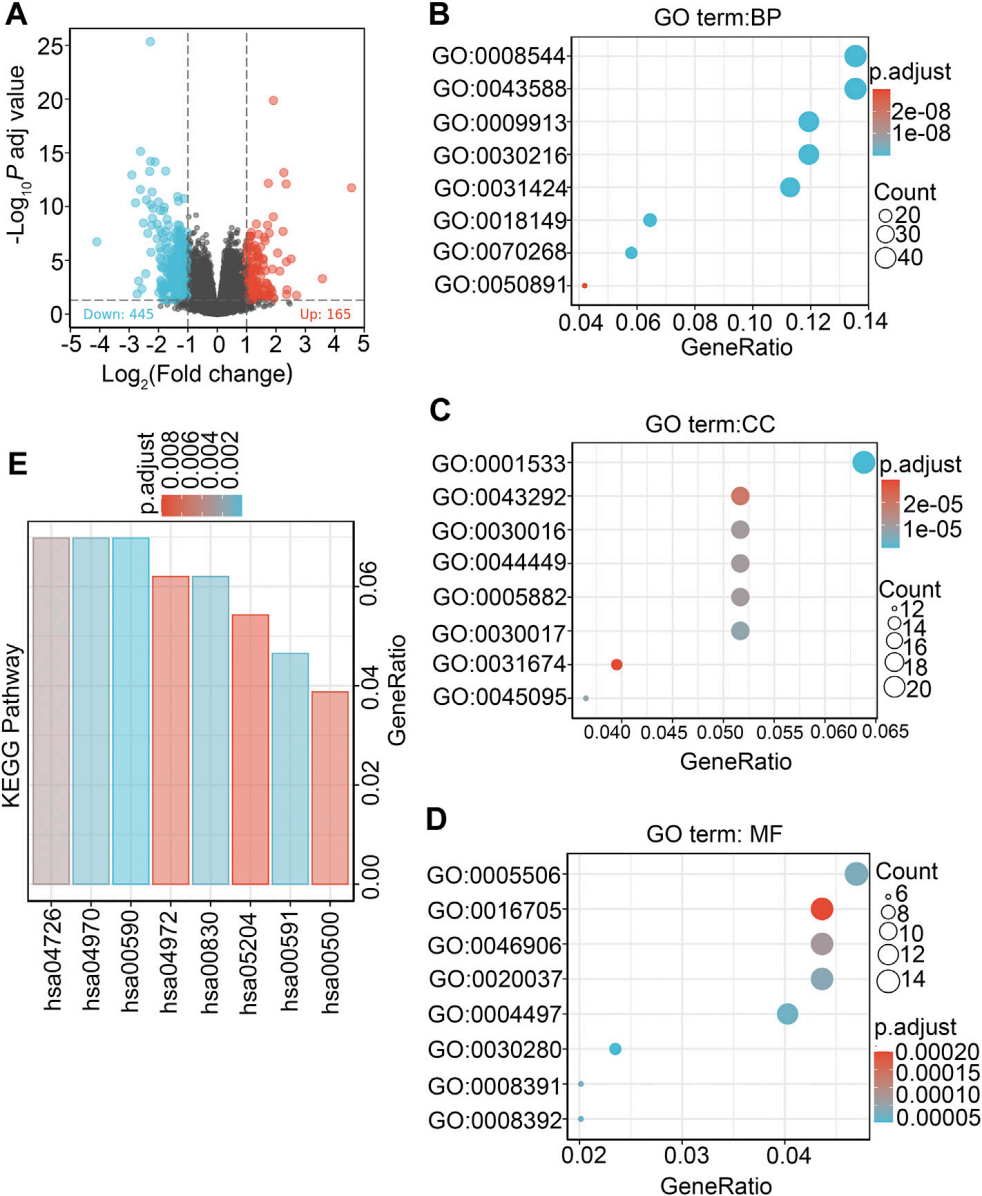
Differentially expressed genes between patients with high and low DDX59-AS1 expression and functional enrichment analysis. **(A)** Volcano plot of differentially expressed genes between the high and low DDX59-AS1 expression groups. Normalized expression levels are shown in descending order from green to red; **(B)** Enriched GO terms in the biological process category; **(C)** Enriched GO terms in the cellular component category; **(D)** Enriched GO terms in the molecular function category. The *x*-axis represents the proportion of differentially expressed genes (DEGs) and the *y*-axis represents different categories. Blue and red tones represent adjusted *p* values from 0.0 to 0.05, respectively, and different circle sizes represent the number of DEGs; **(E)** Enriched KEGG terms in the biological process category.

Data from TCGA were analyzed in DSEeq2 software based on R. As indicated by the volcano plot (Figure 8A), 610 DEGs were identified by screening, with 165 upregulated molecules (logFC>1, padj<0.05) and 445 downregulated molecules (locFC < −1, padj<0.05) (Supplementary Table S2).

### Functional annotation of DDX59-AS1 related DEG in OSCC

**FIGURE 9:**
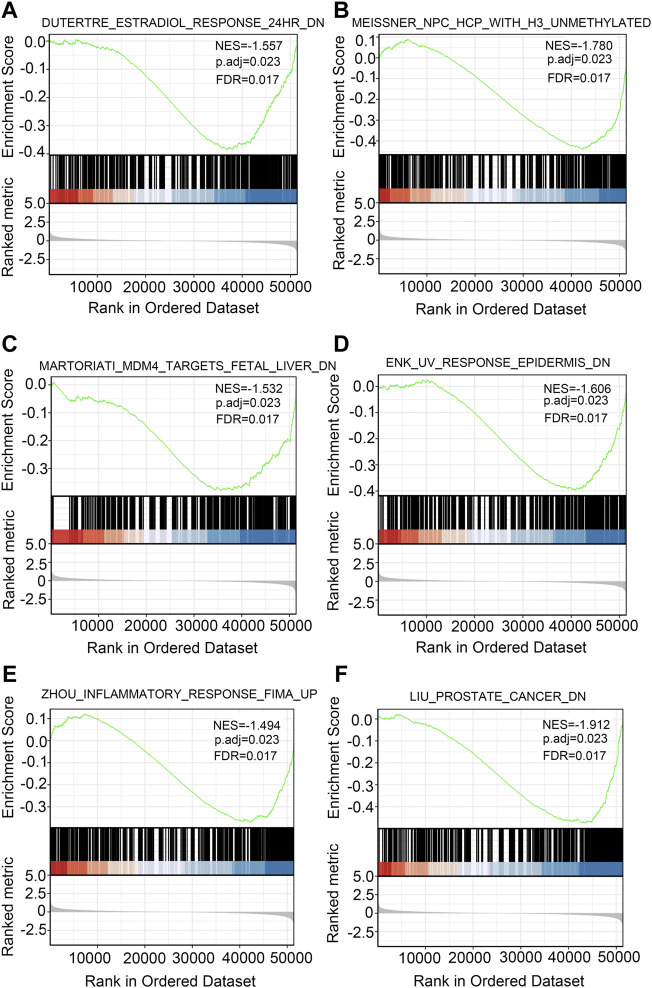
GSEA in OSCC. Several pathways were differentially enriched in OSCC patients according to high and low DDX59-AS1 expression.

To explore the biological functions of the DEGs in high and low DDX59-AS1 expression groups, GO and KEGG analysis were performed with the Clusterprofiler package (Qiu et al., 2019). In OSCC, several enrichments were detected in GO analysis, including in biological process (BP), cellular components (CC), and molecular function (MF), as well as in KEGG pathway enrichment analysis. As of BP, keratineocyte differentiation, epidermal cell differentiation and keratinization were the most significant enrichments, accompanied by the notable enrichments in myogenic fibers, contractile fiber and keratinized envelope on the CC level; analysis in MF highlighted significant enrichment in the oxidoreductase activity, tetrapyrrole binding and iron ion binding (Supplementary Table S3–5). On the other hand, KEGG pathway analysis revealed that DDX59-AS1 DEGs were involved in retinol metabolism, arachidonic acid metabolism, serotonin synapse, salivary secretion, pancreatic secretion, and chemical carcinogenesis (Figures 8B–E) (Supplementary Table S6). Meanwhile, based on the significant difference (padj <0.05, FDR <0.25) in MsigDB Collection [c2.cgp.v7.0.symbols.gmt (Curated)], GSEA analysis identified significant difference in several pathways between the high and low DDX59-AS1 expression groups, including Dutertre estradiol response 24 h dn, Estradiol response 24 h dn, Meissner npc hcp with H3 Unmethylated, Martoriati M4 Targets fetal liver dn, Enk uv response Epidermis dn, Zhou inflammatory response fima up, Liu prostate cancer dn (Montpetit et al., 2011; Yang et al., 2018) (Figure 9). In addition, a PPI network for the DEGs was constructed using the STRING database (You et al., 2017) (Supplementary Figure S2).

### Relationship between DDX59-AS1 and immune infiltration

**FIGURE 10:**
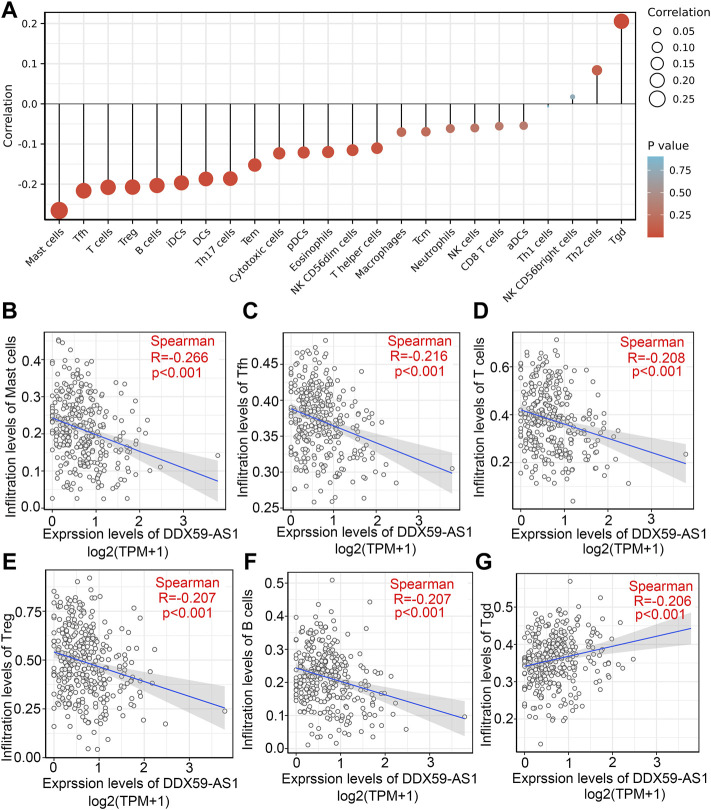
The correlation of DDX59-AS1 expression with immune infiltration level in OSCC. **(A)** Correlations between the relative abundance of 24 immune cells and DDX59-AS1 expression levels. The size of the dots represents the absolute Spearman’s correlation coefficient values; **(B–F)** DDX59-AS1 expression significantly negatively correlates levels of Mast cells, Tfh, T cells, Treg, and B cells; **(G)** DDX59-AS1 expression significantly positively correlates levels of Tgd.

ssGSEA was used to analyze the infiltration of 24 types of immune cells in OSCC based on selected marker genes; the relationship between DDX59-AS1 and these cells were analyzed *via* Spearman’s rank correlation coefficient (Salpietro et al., 2018). As shown in Figure 10A, the expression of DDX59-AS1 was negatively correlated with Mast cells, Tfh, T cells, Treg, and B cells (*p* < 0.01), and positively correlated with Tgd infiltration (*p* < 0.01). The correlations between DDX59-AS1 with these six types of cells were further investigated. The difference in infiltration levels of Mast cells, Tfh, T cells, Treg and B cells was of statistical significance as validated by Wilcoxon rank sum test (Figures 10B–G). In addition, we also used the CIBERSORT algorithm to calculate the correlation between immune cells and OSCC tissue expression profile data and the degree of correlation between DDX59-AS1 and immune cells (Supplementary Figure S3,4). These results provide further evidence that DDX59-AS1 expression is associated with the extent of immune cell infiltration in OSCC.

The estimate package uses the unique properties of the transcriptional profile of cancer samples to infer the content of tumor cells and the different infiltrating normal cells. It mainly uses RNA-seq data to calculate the immune and stromal scores of the sample, and then evaluate the purity of the tumor. The results showed statistically significant differences between the different groups of the samples by the three scoring methods: StromalScore, ImmuneScore, and ESTIMATEScore (Supplementary Figure S5).

## Discussion

LncRNAs are produced by bidirectional transcription of genes and belong to a large heterogeneous group of ncRNAs based on the size of the transcript. Different classes of lncRNAs are involved in many gene expression processes, including regulation of chromatin activity and transcription in the nucleus, as well as RNA maturation, stability, translation and post-translational modifications in the cytoplasm. Notably, different lncRNAs are present in different cells, tissues and diseases in a highly specific manner, suggesting that lncRNAs are potential biomarkers for different diseases (Liang et al., 2021). In fact, lncRNAs play an increasingly important role in the progression of OSCC.

The DEAD-box decarboxylase 5/DEAD-box protein 2 (DDX5/Dbp2) family is a member of DexDH/H-box-containing ATP-dependent RNA decarboxylase family. These proteins play a variety of roles, including RNA metabolism, RNA stability, transport, biogenesis, cell differentiation, and metabolism (Qiu et al., 2019). As a member of the DDX5.Dbp2 subfamily, DDX59 has nine highly conserved sequences, the most characteristic regions including aspartate-glutamate-alanine-aspartate (D-E-A-D), which has been reported to be involved in malignancies, neurological development, and developmental delay (Montpetit et al., 2011; You et al., 2017; Salpietro et al., 2018; Yang et al., 2018). However, the involvement of DDX59-AS1 in OSCC remained exploring in future studies. As effective prognostic biomarkers acting as an essential component of personalized medicine and precision medicine which provide important information about the clinical outcome of cancer treatment, our results consistently suggest DDX59-AS1 expression as a reliable predictor of clinical outcome in OSCC (Liang et al., 2021).

In this research, the first study of DDX59-AS1 in OSCC, the process of screening for DDX59-AS1 is as follows: 1) several differentially expressed genes were screened by TCGA RNA-sequencing; 2) Through prognostic analysis, it is found that DDX59-AS1 has a good prognosis; by looking for the molecules related to OSCC, it is found that DDX59-AS1 has not been reported in OSCC, which is an original research; 3) By performing correlation analysis, DDX59-AS1 was found to be positive in clinical correlation analysis, 4) By looking up the literature related to DDX59-AS1 and consulting the gene database of Genecards/Pubmed, we found that DDX59-AS1 is highly expressed in pan-cancer, especially in lung cancer, but there is no research in OSCC, so we chose this gene for research, and the potential molecular and mechanism of action of this gene in OSCC were screened.

The present study is the first study on DDX59-AS1. We found for the first time that DDX59-AS1 had a significantly high expression in the OSCC and the pan-cancer tissue compared to that of the normal tissue. Having an AUC value of 0.732 of the ROC curves to distinguish OSCC, DDX59-AS1 was identified as a convincing biomarker for the diagnosis of OSCC. By mining the TCGA data, we confirmed the predictive value of DDX59-AS1 for overall and disease-specific survival of OSCC patients. As survival rate was lower in patients with high DDX59-AS1 expression in the first 150 months, the nomogram suggested the high expression of DDX59-AS1 is associated with a decreased overall survival. Therefore, we speculate that DDX59-AS1 is also a potential biomarker for OSCC.

The tumor microenvironment (TME) is critical in the mediation of physiological processes of cancer cells, in which tumor-infiltrating immune cells are an indispensable component. In this study, we sought to explore the potential relationship between DDX59-AS1 expression and immune cell infiltration. DDX59-AS1 was negatively correlated with Mast cells, T cells, Treg, and, B cells. Mast cells are typical precursor cells of the immune system. They accumulated in the tumor stroma of different human cancer types and played a multifaceted role in the TME by regulating various biological events involved in tumor, such as cell proliferation and survival, angiogenesis, invasion, and metastasis (Aponte-López and Muñoz-Cruz, 2020). While an increased number of mast cells in the TME has been reported to be associated with tumorigenesis, it is notable that a decrease or loss of mast cells within tumors was associated with more severe diseases (Hempel Sullivan et al., 2021). A variety of cell types contained in TME were involved in various biological processes that promote or inhibit tumor progression, among which immunosuppressive cells could contribute to T cell dysfunction. The suppressor cells include regulatory T cells (Treg cells), tumor-associated macrophages (TAM), myeloid-derived suppressor cells (MDSC), cancer-associated fibroblasts and adipocytes, and endothelial cells (Xia et al., 2019). B cells are the primary effector cells of humoral immunity, which inhibit tumor progression by secreting immunoglobulins, promoting T cell responses, and directly killing cancer cells. Given these properties, their antitumor immune response in the TME has attracted significant interest (Tokunaga et al., 2019).

Here, we identified the clinical significance of DDX59-AS1 in OSCC, the high expression of which was related to an unfavorable prognosis of the disease in the population featuring T2-4 stage, clinical stage II-IV, black or African American and less than or equal to 60 years old. In this research, DDX59-AS1 is the first study in OSCC. Currently, only the differential analysis of this gene is available in the TCGA database, but there is no differential expression of this gene in the GEO database. In future studies, we will further verify the expression of DDX59-AS1 *in vivo* and *in vitro*. At the same time, the dynamic nomogram is drawn and analyzed. In conclusion, DDX59-AS1 could be a potential prognostic indicator and therapeutic target for OSCC.

Despite the discovery and validation of the diagnostic and prognostic value of DDX59-AS1 in OSCC, there are still some limitations in this study. First, the sample information is incomplete, and the specific pathogenesis and molecular targets need to be further verified. In conclusion, our study revealed for the first time the prognostic value of DDX59-AS1 in OSCC. Our study demonstrates that DDX59-AS1 is a potential biomarker to predict treatment outcome and prognosis in OSCC patients. However, further experimental validation is required to elucidate the biological effects and underlying mechanisms of DDX59-AS1.

## Data Availability

The datasets presented in this study can be found in online repositories. The names of the repository/repositories and accession number(s) can be found in the article/Supplementary Material.

## Supplementary material

SUPPLEMENTARY FIGURE S1 Disease-Specific Survival for subgroup analyses. **(A)** T stage; **(B)** N stage; **(C)** M stage; **(D)** Clinical stage; **(E)** Primary therapy outcome; **(F)** Gender; **(G)** Race; **(H)** Age; **(I)** Histologic grade; **(J)** Smoker; **(K)** Alcohol; **(L)** Perineural invasion; **(M)** Lymph vascular invasion; **(N)** TP53 status; **(O)** PIK3CA status.

SUPPLEMENTARY FIGURE S2 Construction of protein-protein interaction network of DDX59-AS1 and its co-expressed genes.

SUPPLEMENTARY FIGURE S3 Immune Infiltration Analysis of Oral Squamous Cell Carcinoma Dataset TCGA-OSCC (CIBERSORT). **(A)** Histogram display of immune infiltration results of 22 types of immune cells in the TCGA-OSCC dataset; **(B)** Group comparison chart of immune infiltration results of TCGA-OSCC dataset; **(C)** Heat map display of the correlation analysis results of 22 immune cells; **(D)** Lollipop chart display of the correlation analysis results between the target gene DDX59-AS1 and the infiltration abundance of 22 types of immune cells.

SUPPLEMENTARY FIGURE S4 Correlation analysis of target gene DDX59-AS1 and immune cell infiltration abundance. **(A)** Target gene DDX59-AS1 and immune cells **(A)** B cells memory; **(B)** Dendritic cells resting; **(C)** Eosinophils; **(D)** Mast cells activated; **(E)** Mast cells resting; **(F)** NK cells resting; **(G)** Results of correlation analysis between T cells CD8; **(H)** T cells follicular helper, and **(I)** T cells regulatory (Tregs) infiltration abundance.

SUPPLEMENTARY FIGURE S5 Immune score (estimate) of OSCC dataset TCGA. **(A)** StromalScore; **(B)** ImmuneScore; **(C)** ESTIMATEScore.

SUPPLEMENTARY TABLE S1 Disease-Specific survival prognostic performance of DDX59-AS1 on clinical outcomes in OSCC patient subgroups by Cox regression analysis.

SUPPLEMENTARY TABLE S2 Differential gene expression profile in OSCC patients stratified by DDX59-AS1 levels.

SUPPLEMENTARY TABLE S3 Biological process of Gene Ontology enrichment analysis.

SUPPLEMENTARY TABLE S4 Cellular components of Gene Ontology enrichment analysis.

SUPPLEMENTARY TABLE S5 Molecular function of Gene Ontology enrichment analysis.

SUPPLEMENTARY TABLE S6 KEGG pathway enrichment analysis.

SUPPLEMENTARY TABLE S7 Protein to protein interactions of DDX59-AS1.
