# Supplementary material for: DDX59-AS1 is a prognostic biomarker and correlated with immune infiltrates in OSCC
Source: Front Genet. 2022 Aug 23;13:892727. doi: 10.3389/fgene.2022.892727 (PMC9447487; doi:10.3389/fgene.2022.892727)
Supplement: Supplementary file 1 [file Table1.docx]

| Characteristics | N (%) | HR(95% CI) | P value |
| --- | --- | --- | --- |
| T stage |  |  |  |
| T1 | 20 (7) | 3.068(0.429-21.917) | 0.264 |
| T2&T3&T4 | 283 (93) | 1.633(1.063-2.510) | 0.025 |
| Clinical stage |  |  |  |
| Stage I | 11 (4) | 2.481(0.222-27.770) | 0.461 |
| Stage II | 72 (24) | 1.330(0.535-3.311) | 0.539 |
| Stage III | 62 (20) | 2.578(0.828-8.029) | 0.102 |
| Stage IV | 158 (52) | 1.747(1.013-3.013) | 0.045 |
| Gender |  |  |  |
| Female | 95 (30) | 2.175(0.928-5.099) | 0.074 |
| Male | 218 (70) | 1.684(1.038-2.731) | 0.035 |
| Age |  |  |  |
| <=60 | 152 (49) | 1.550(0.845-2.842) | 0.157 |
| >60 | 161 (51) | 2.042(1.126-3.704) | 0.019 |
| Smoker |  |  |  |
| No | 83 (27) | 1.435(0.618-3.331) | 0.400 |
| Yes | 225 (73) | 1.982(1.216-3.231) | 0.006 |
| Alcohol history |  |  |  |
| No | 98 (32) | 2.382(1.008-5.627) | 0.048 |
| Yes | 208 (68) | 1.622(1.000-2.630) | 0.050 |
| Lymphovascular invasion |  |  |  |
| No | 158 (70) | 3.721(1.828-7.573) | <0.001 |
| Yes | 68 (30) | 0.894(0.399-2.003) | 0.786 |
| Perineural invasion |  |  |  |
| No | 111 (47) | 2.165(0.908-5.166) | 0.082 |
| Yes | 126 (53) | 1.729(0.959-3.117) | 0.069 |
| TP53 status |  |  |  |
| WT | 101 (33) | 1.823(0.843-3.941) | 0.127 |
| Mut | 209 (67) | 1.827(1.101-3.032) | 0.020 |
| PIK3CA status |  |  |  |
| WT | 261 (84) | 2.097(1.298-3.390) | 0.002 |
| Mut | 49 (16) | 1.082(0.428-2.732) | 0.868 |
| Primary therapy outcome |  |  |  |
| PD&SD&PR | 41 (15) | 1.477(0.674-3.236) | 0.330 |
| CR | 229 (85) | 1.865(0.973-3.576) | 0.060 |
| Histologic grade |  |  |  |
| G1&G2 | 241 (78) | 1.990(1.212-3.266) | 0.007 |
| G3&G4 | 67 (22) | 1.219(0.553-2.688) | 0.624 |
| Race |  |  |  |
| Asian&Black or African American | 30 (10) | 3.495(0.442-27.649) | 0.236 |
| White | 272 (90) | 1.575(1.014-2.447) | 0.043 |
| N stage |  |  |  |
| N0&N1 | 208 (70) | 1.652(0.955-2.856) | 0.072 |
| N2&N3 | 91 (30) | 2.100(1.070-4.121) | 0.031 |

HR, hazard ratio; CI, confidence interval; WT, wild type; Mut, mutation.
