# Supplementary figures and images for: DDX59-AS1 is a prognostic biomarker and correlated with immune infiltrates in OSCC
Source: Front Genet. 2022 Aug 23;13:892727. doi: 10.3389/fgene.2022.892727 (PMC9447487; doi:10.3389/fgene.2022.892727)

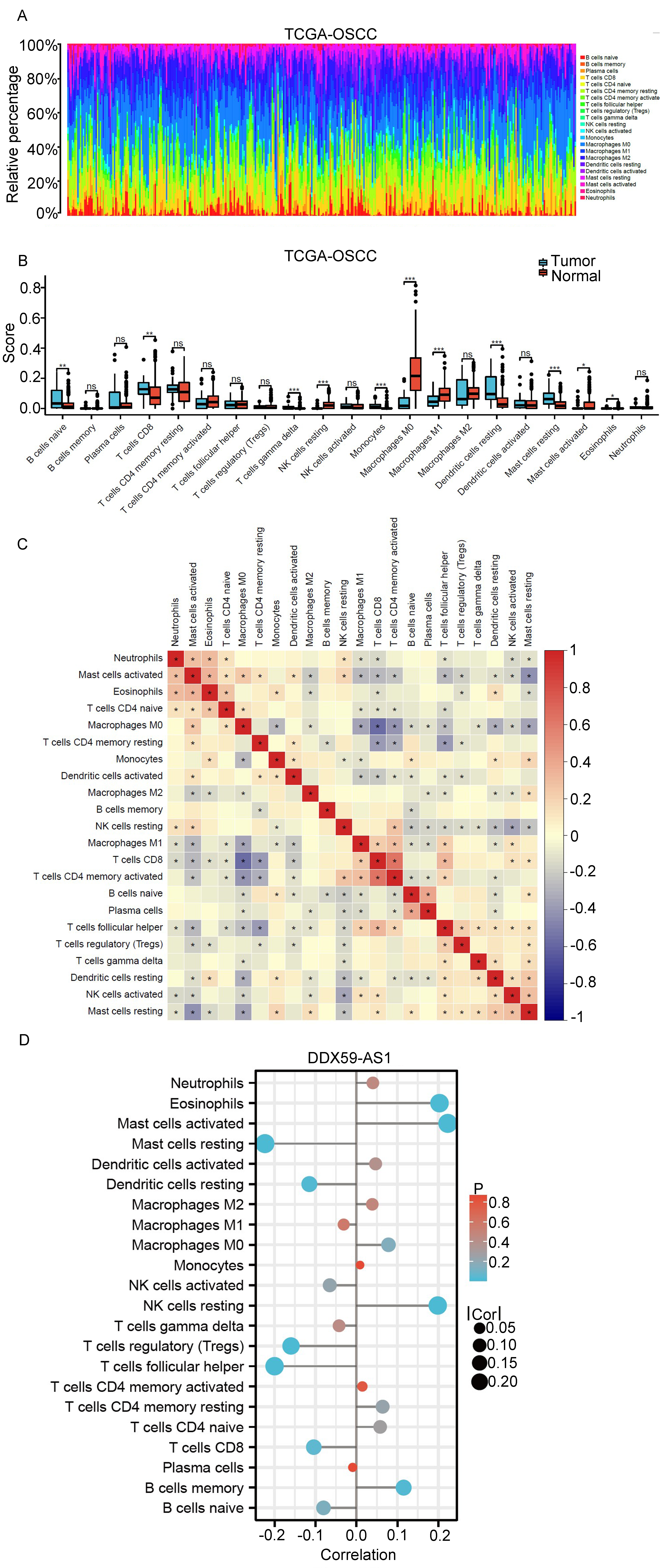

Supplement: Supplementary file 2 [file Image3.TIF]

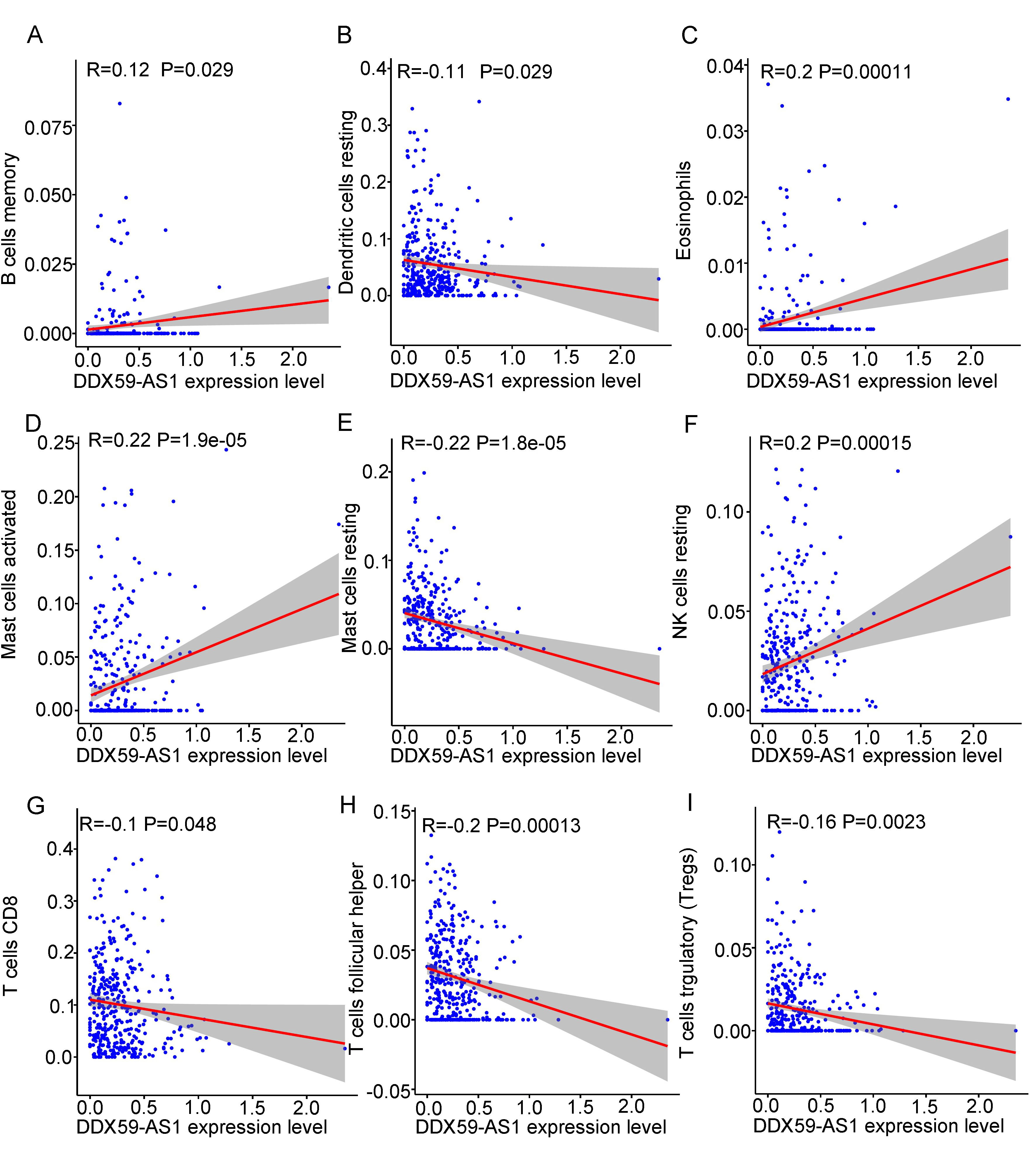

Supplement: Supplementary file 3 [file Image4.TIF]

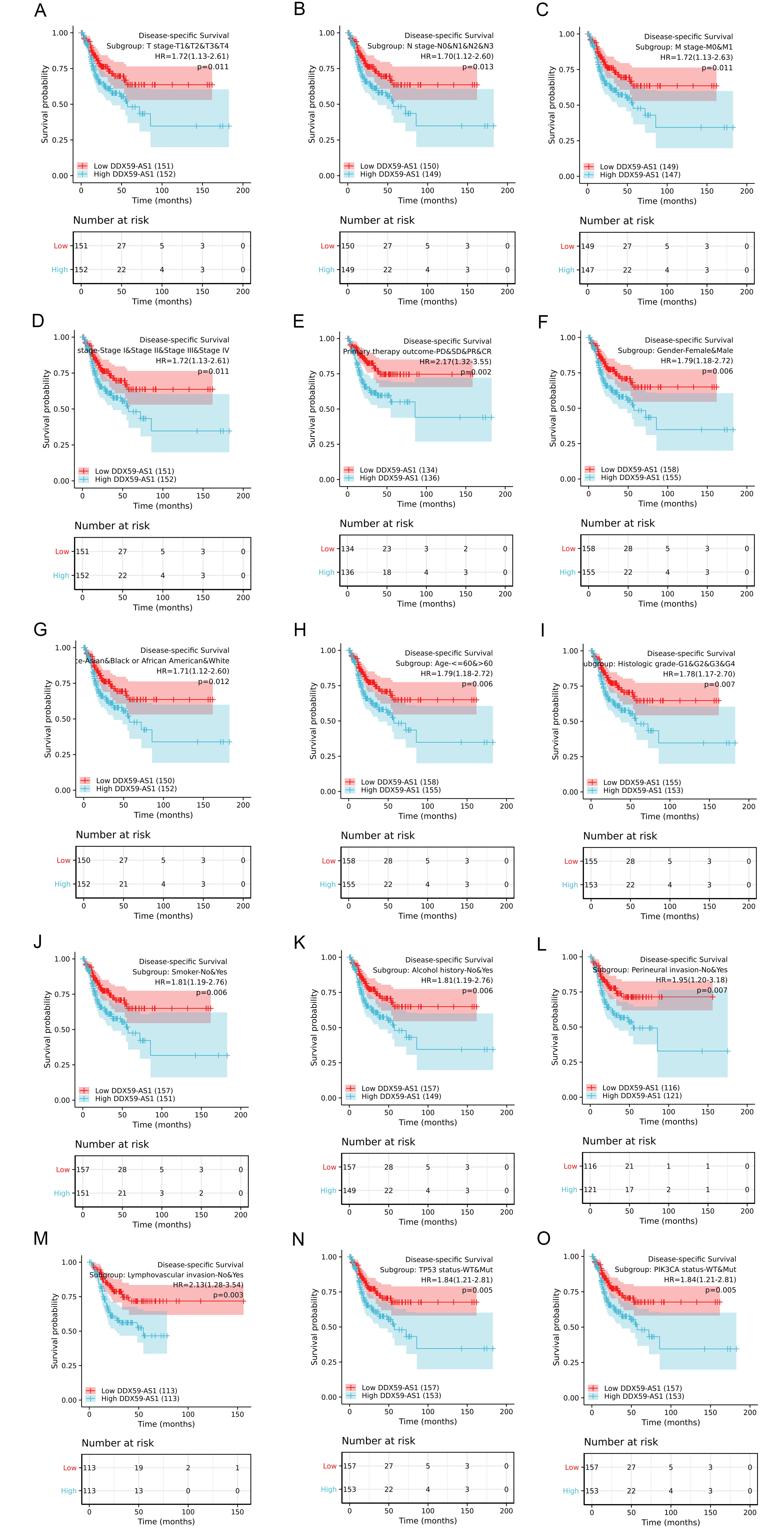

Supplement: Supplementary file 5 [file Image1.TIF]

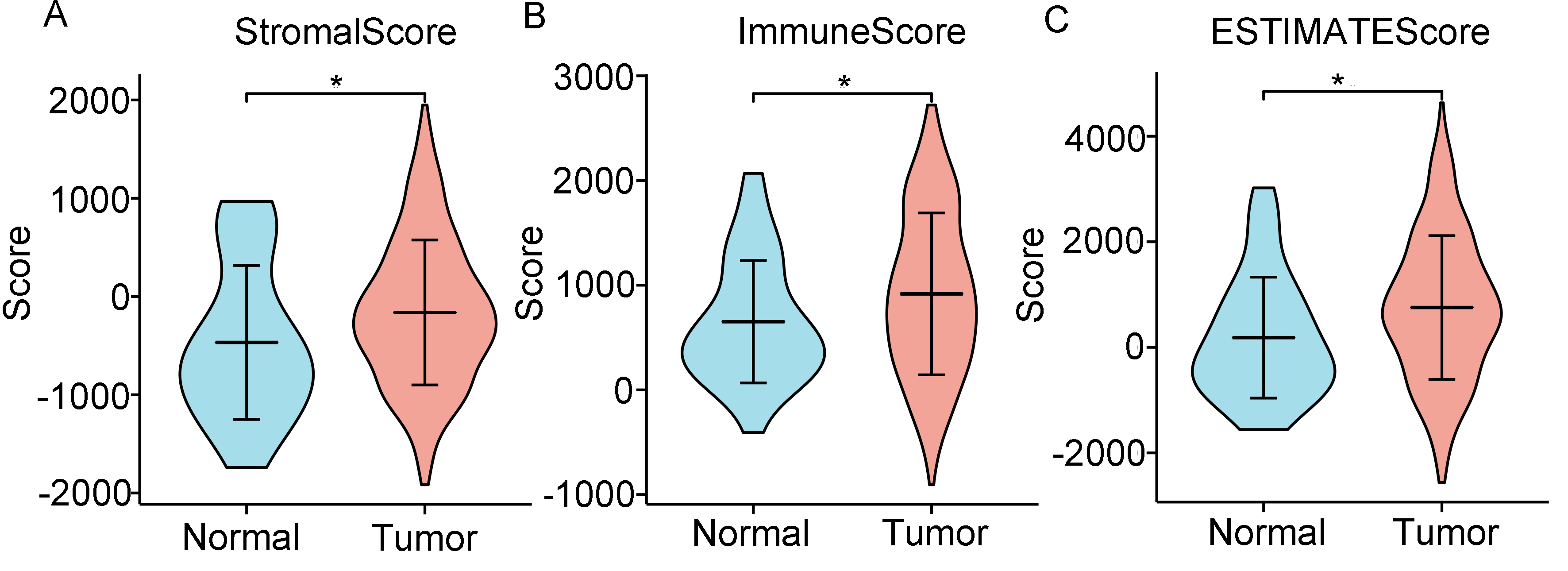

Supplement: Supplementary file 11 [file Image5.TIF]

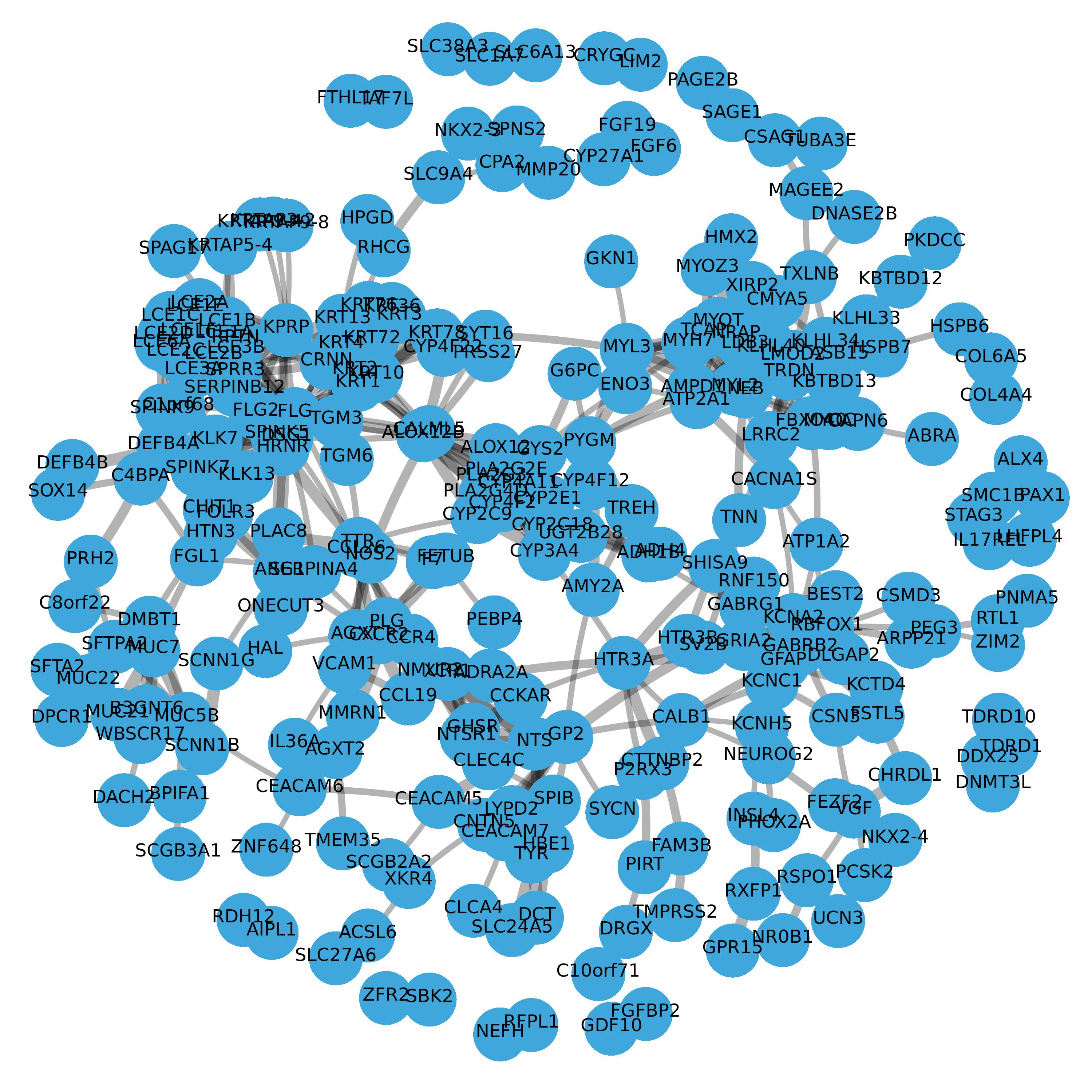

Supplement: Supplementary file 12 [file Image2.TIFF]
