# Supplementary material for: DDX59-AS1 is a prognostic biomarker and correlated with immune infiltrates in OSCC
Source: Front Genet. 2022 Aug 23;13:892727. doi: 10.3389/fgene.2022.892727 (PMC9447487; doi:10.3389/fgene.2022.892727)
Supplement: Supplementary file 4 [file Table7.docx]

| Characteristics | Total(N) | HR(95% CI) Univariate analysis | P value Univariate analysis | HR(95% CI) Multivariate analysis | P value Multivariate analysis |
| --- | --- | --- | --- | --- | --- |
| T stage (T3&T4 vs. T1&T2) | 303 | 1.788(1.129-2.833) | 0.013 | 1.793(0.915-3.512) | 0.089 |
| N stage (N1&N2&N3 vs. N0) | 299 | 1.570(1.037-2.376) | 0.033 | 0.974(0.538-1.763) | 0.932 |
| M stage (M1 vs. M0) | 296 | 3.922(0.542-28.377) | 0.176 |  |  |
| Clinical stage (Stage III&Stage IV vs. Stage I&Stage II) | 303 | 1.336(0.826-2.159) | 0.238 |  |  |
| Primary therapy outcome (CR vs. PD&SD&PR) | 270 | 0.082(0.050-0.136) | <0.001 | 0.125(0.070-0.224) | <0.001 |
| Histologic grade (G3&G4 vs. G1&G2) | 308 | 1.327(0.837-2.104) | 0.229 |  |  |
| Gender (Male vs. Female) | 313 | 1.309(0.821-2.089) | 0.258 |  |  |
| Race (White vs. Asian&Black or African American) | 302 | 0.606(0.313-1.172) | 0.136 |  |  |
| Age (>60 vs. <=60) | 313 | 1.170(0.776-1.763) | 0.454 |  |  |
| Smoker (Yes vs. No) | 308 | 1.196(0.740-1.933) | 0.465 |  |  |
| Alcohol history (Yes vs. No) | 306 | 1.482(0.922-2.384) | 0.105 |  |  |
| Perineural invasion (Yes vs. No) | 237 | 2.160(1.302-3.581) | 0.003 | 1.829(0.978-3.420) | 0.059 |
| Lymphovascular invasion (Yes vs. No) | 226 | 1.591(0.963-2.630) | 0.070 | 1.173(0.654-2.105) | 0.593 |
| TP53 status (Mut vs. WT) | 310 | 1.117(0.712-1.753) | 0.630 |  |  |
| PIK3CA status (Mut vs. WT) | 310 | 1.219(0.727-2.044) | 0.452 |  |  |
| DDX59-AS1 (High vs. Low) | 313 | 1.791(1.177-2.723) | 0.006 | 2.034(1.150-3.596) | 0.015 |
