# Supplementary material for: DDX59-AS1 is a prognostic biomarker and correlated with immune infiltrates in OSCC
Source: Front Genet. 2022 Aug 23;13:892727. doi: 10.3389/fgene.2022.892727 (PMC9447487; doi:10.3389/fgene.2022.892727)
Supplement: Supplementary file 6 [file Table2.docx]

| gene_id | baseMean | log2FoldChange | lfcSE | stat | pvalue | padj | gene_name | gene_biotype | cor_pvalue | correlation |
| --- | --- | --- | --- | --- | --- | --- | --- | --- | --- | --- |
| ENSG00000186847 | 947184.72 | 0.050894779 | 0.16201914 | 0.31412819 | 7.534237e-01 | 8.644043e-01 | KRT14 | protein_coding | 8.051297e-01 | 0.013611484 |
| ENSG00000186081 | 852885.92 | -0.117340795 | 0.10773056 | -1.08920623 | 2.760630e-01 | 4.698800e-01 | KRT5 | protein_coding | 3.057954e-01 | -0.056457447 |
| ENSG00000205420 | 741547.82 | -0.422278982 | 0.13106343 | -3.22194369 | 1.273242e-03 | 1.086411e-02 | KRT6A | protein_coding | 5.170299e-03 | -0.153364484 |
| ENSG00000128422 | 564530.60 | 0.158866434 | 0.14028815 | 1.13242942 | 2.574539e-01 | 4.493940e-01 | KRT17 | protein_coding | 5.423057e-02 | 0.105909936 |
| ENSG00000186832 | 478640.58 | -0.430945163 | 0.18148986 | -2.37448613 | 1.757340e-02 | 7.169460e-02 | KRT16 | protein_coding | 3.160404e-03 | -0.161781486 |
| ENSG00000075624 | 353904.47 | 0.095414270 | 0.05345697 | 1.78487987 | 7.428080e-02 | 1.984589e-01 | ACTB | protein_coding | 6.158885e-03 | 0.150272680 |
| ENSG00000198804 | 349311.82 | -0.187929608 | 0.08881273 | -2.11602098 | 3.434302e-02 | 1.159254e-01 | MT-CO1 | protein_coding | 2.491752e-02 | -0.123264645 |
| ENSG00000108821 | 314033.72 | -0.365476218 | 0.17182999 | -2.12696403 | 3.342307e-02 | 1.137033e-01 | COL1A1 | protein_coding | 2.868385e-01 | -0.058713839 |
| ENSG00000163220 | 256278.31 | -0.407533036 | 0.17462804 | -2.33372044 | 1.961037e-02 | 7.758267e-02 | S100A9 | protein_coding | 1.869084e-05 | -0.232887527 |
| ENSG00000198886 | 251599.67 | -0.079849485 | 0.09424786 | -0.84722859 | 3.968677e-01 | 5.919708e-01 | MT-ND4 | protein_coding | 6.343393e-01 | -0.026237648 |
| ENSG00000210082 | 241878.89 | -0.147845620 | 0.10181315 | -1.45212692 | 1.464663e-01 | 3.130606e-01 | MT-RNR2 | Mt_rRNA | 3.102261e-01 | -0.055943525 |
| ENSG00000185479 | 240798.56 | -0.524491720 | 0.18046977 | -2.90625800 | 3.657797e-03 | 2.308559e-02 | KRT6B | protein_coding | 1.396169e-02 | -0.135009067 |
| ENSG00000184009 | 237989.34 | 0.076502869 | 0.05471236 | 1.39827393 | 1.620308e-01 | 3.337358e-01 | ACTG1 | protein_coding | 2.100418e-03 | 0.168480506 |
| ENSG00000175793 | 228230.56 | 0.097178603 | 0.12709584 | 0.76460883 | 4.445045e-01 | 6.363640e-01 | SFN | protein_coding | 6.034350e-01 | 0.028655034 |
| ENSG00000111640 | 214808.93 | 0.247734103 | 0.07258848 | 3.41285682 | 6.428571e-04 | 6.589332e-03 | GAPDH | protein_coding | 2.036935e-05 | 0.231872589 |
| ENSG00000168542 | 197708.50 | -0.359620359 | 0.18085123 | -1.98848723 | 4.675783e-02 | 1.443179e-01 | COL3A1 | protein_coding | 1.694114e-01 | -0.075705195 |
| ENSG00000096696 | 196245.33 | -0.456550615 | 0.12156688 | -3.75555090 | 1.729606e-04 | 2.495483e-03 | DSP | protein_coding | 1.355639e-02 | -0.135583714 |
| ENSG00000198938 | 187804.86 | -0.087637660 | 0.08990530 | -0.97477748 | 3.296706e-01 | 5.271224e-01 | MT-CO3 | protein_coding | 3.776333e-01 | -0.048649011 |
| ENSG00000156508 | 179762.59 | 0.006105456 | 0.05681956 | 0.10745342 | 9.144293e-01 | 9.578607e-01 | EEF1A1 | protein_coding | 1.004227e-01 | 0.090453893 |
| ENSG00000164692 | 170247.38 | -0.352561322 | 0.17245749 | -2.04433760 | 4.092021e-02 | 1.315441e-01 | COL1A2 | protein_coding | 1.841936e-01 | -0.073169836 |
| ENSG00000166710 | 168936.94 | 0.102917556 | 0.08660707 | 1.18832743 | 2.347044e-01 | 4.236372e-01 | B2M | protein_coding | 9.301422e-02 | 0.092476985 |
| ENSG00000198712 | 164251.76 | 0.015634077 | 0.08670563 | 0.18031212 | 8.569075e-01 | 9.259923e-01 | MT-CO2 | protein_coding | 5.776099e-01 | 0.030717836 |
| ENSG00000143546 | 160446.83 | -0.501230700 | 0.18545534 | -2.70270298 | 6.877817e-03 | 3.669313e-02 | S100A8 | protein_coding | 1.862507e-04 | -0.204009948 |
| ENSG00000234745 | 154696.26 | 0.322847393 | 0.10886747 | 2.96550824 | 3.021832e-03 | 2.008730e-02 | HLA-B | protein_coding | 2.792365e-03 | 0.163837670 |
| ENSG00000074800 | 143431.17 | 0.214403235 | 0.06099175 | 3.51528284 | 4.392859e-04 | 4.962496e-03 | ENO1 | protein_coding | 4.670025e-06 | 0.248665888 |
| ENSG00000112378 | 141994.29 | -0.355797731 | 0.09373564 | -3.79575722 | 1.471935e-04 | 2.210614e-03 | PERP | protein_coding | 2.271506e-03 | -0.167215391 |
| ENSG00000067225 | 141878.04 | 0.128904811 | 0.06896771 | 1.86906032 | 6.161442e-02 | 1.746137e-01 | PKM | protein_coding | 2.134294e-03 | 0.168222717 |
| ENSG00000104419 | 135204.24 | 0.145678521 | 0.12294715 | 1.18488737 | 2.360620e-01 | 4.250224e-01 | NDRG1 | protein_coding | 1.214991e-01 | 0.085281416 |
| ENSG00000170465 | 132107.00 | -0.780731679 | 0.22709425 | -3.43791922 | 5.862025e-04 | 6.158879e-03 | KRT6C | protein_coding | 4.279074e-04 | -0.192514379 |
| ENSG00000173801 | 131400.82 | -0.243860386 | 0.09605863 | -2.53866205 | 1.112773e-02 | 5.170620e-02 | JUP | protein_coding | 1.794959e-02 | -0.130022404 |
| ENSG00000115414 | 130253.61 | -0.100361176 | 0.19537894 | -0.51367449 | 6.074796e-01 | 7.652803e-01 | FN1 | protein_coding | 8.258531e-01 | 0.012139047 |
| ENSG00000171401 | 129143.96 | -2.041511860 | 0.29473150 | -6.92668365 | 4.308205e-12 | 4.344025e-09 | KRT13 | protein_coding | 5.584788e-07 | -0.270963910 |
| ENSG00000198727 | 125032.23 | -0.162145030 | 0.10061254 | -1.61157879 | 1.070536e-01 | 2.543100e-01 | MT-CYB | protein_coding | 3.331645e-02 | -0.117019650 |
| ENSG00000196754 | 120241.57 | 0.493108363 | 0.13224868 | 3.72864491 | 1.925122e-04 | 2.719233e-03 | S100A2 | protein_coding | 7.071979e-04 | 0.185241044 |
| ENSG00000133112 | 118800.55 | 0.137952332 | 0.07162577 | 1.92601547 | 5.410244e-02 | 1.597210e-01 | TPT1 | protein_coding | 3.098586e-02 | 0.118603278 |
| ENSG00000211896 | 118451.89 | 0.057014382 | 0.23877759 | 0.23877610 | 8.112792e-01 | 8.995552e-01 | IGHG1 | IG_C_gene | 7.252580e-03 | -0.147333100 |
| ENSG00000167658 | 115625.98 | -0.002330839 | 0.05734647 | -0.04064485 | 9.675790e-01 | 9.850869e-01 | EEF2 | protein_coding | 3.545292e-01 | 0.051048692 |
| ENSG00000161016 | 114120.60 | 0.445391131 | 0.08956958 | 4.97257128 | 6.607067e-07 | 4.449809e-05 | RPL8 | protein_coding | 5.649693e-07 | 0.270847922 |
| ENSG00000100345 | 113795.08 | -0.002681183 | 0.07603172 | -0.03526401 | 9.718692e-01 | 9.865718e-01 | MYH9 | protein_coding | 5.553515e-01 | 0.032530627 |
| ENSG00000124102 | 110311.20 | -0.440418341 | 0.23148237 | -1.90259992 | 5.709277e-02 | 1.653016e-01 | PI3 | protein_coding | 5.682087e-02 | -0.104799031 |
| ENSG00000081277 | 106760.81 | -0.272056583 | 0.11060787 | -2.45964937 | 1.390728e-02 | 6.072026e-02 | PKP1 | protein_coding | 3.815954e-02 | -0.114009418 |
| ENSG00000135046 | 97703.46 | -0.356572809 | 0.13175060 | -2.70642262 | 6.801243e-03 | 3.641121e-02 | ANXA1 | protein_coding | 4.538672e-02 | -0.110069792 |
| ENSG00000196924 | 96823.05 | 0.085940653 | 0.09200655 | 0.93407104 | 3.502672e-01 | 5.474681e-01 | FLNA | protein_coding | 1.134035e-01 | 0.087177930 |
| ENSG00000164924 | 96459.23 | -0.012236315 | 0.06952544 | -0.17599768 | 8.602958e-01 | 9.278171e-01 | YWHAZ | protein_coding | 9.261229e-01 | 0.005115889 |
| ENSG00000206503 | 96075.48 | 0.325133056 | 0.10237246 | 3.17598162 | 1.493304e-03 | 1.223878e-02 | HLA-A | protein_coding | 1.969275e-03 | 0.169515630 |
| ENSG00000198899 | 95903.22 | -0.141357100 | 0.09464694 | -1.49351999 | 1.353011e-01 | 2.968734e-01 | MT-ATP6 | protein_coding | 2.435825e-01 | -0.064270350 |
| ENSG00000124942 | 95732.38 | -0.279314496 | 0.09033762 | -3.09189552 | 1.988828e-03 | 1.502695e-02 | AHNAK | protein_coding | 2.230764e-02 | -0.125577294 |
| ENSG00000019582 | 94774.44 | -0.101965174 | 0.14956722 | -0.68173477 | 4.954067e-01 | 6.795211e-01 | CD74 | protein_coding | 1.009766e-01 | -0.090307460 |
| ENSG00000106211 | 94516.06 | -0.164764036 | 0.10326051 | -1.59561523 | 1.105747e-01 | 2.595818e-01 | HSPB1 | protein_coding | 1.135363e-01 | -0.087145996 |
| ENSG00000182718 | 93928.78 | 0.161796244 | 0.06657182 | 2.43040144 | 1.508211e-02 | 6.420769e-02 | ANXA2 | protein_coding | 1.351194e-04 | 0.208280331 |
