# Supplementary material for: DDX59-AS1 is a prognostic biomarker and correlated with immune infiltrates in OSCC
Source: Front Genet. 2022 Aug 23;13:892727. doi: 10.3389/fgene.2022.892727 (PMC9447487; doi:10.3389/fgene.2022.892727)
Supplement: Supplementary file 7 [file Table5.docx]

| ID | Description | GeneRatio | BgRatio | pvalue | p.adjust | qvalue | geneID | Count |
| --- | --- | --- | --- | --- | --- | --- | --- | --- |
| GO:0030280 | structural constituent of epidermis | 7/298 | 16/17697 | 3.595018e-09 | 1.650113e-06 | 1.422870e-06 | KRT1/KRT10/FLG/KRT2/FLG2/HRNR/KRT36 | 7 |
| GO:0004497 | monooxygenase activity | 12/298 | 99/17697 | 1.043767e-07 | 2.305030e-05 | 1.987596e-05 | CYP4F22/CYP27A1/CYP2C18/CYP4F12/CYP2E1/NOS2/CYP4F2/CYP2C9/CYP4Z1/CYP3A4/TYR/CYP4A11 | 12 |
| GO:0008392 | arachidonic acid epoxygenase activity | 6/298 | 16/17697 | 1.506556e-07 | 2.305030e-05 | 1.987596e-05 | CYP2C18/CYP4F12/CYP2E1/CYP4F2/CYP2C9/CYP4A11 | 6 |
| GO:0008391 | arachidonic acid monooxygenase activity | 6/298 | 17/17697 | 2.295457e-07 | 2.634037e-05 | 2.271294e-05 | CYP2C18/CYP4F12/CYP2E1/CYP4F2/CYP2C9/CYP4A11 | 6 |
| GO:0005506 | iron ion binding | 14/298 | 152/17697 | 2.947665e-07 | 2.705956e-05 | 2.333309e-05 | ALOX12B/CYP4F22/CYP27A1/ALOX12/CYP2C18/CYP4F12/CYP2E1/CYP4F2/CYP2C9/CYP4Z1/CYP3A4/CYP4Z2P/CYP4A11/FTHL17 | 14 |
| GO:0020037 | heme binding | 13/298 | 135/17697 | 4.703164e-07 | 3.597920e-05 | 3.102438e-05 | CYP4F22/CYP27A1/CYP2C18/CYP4F12/CYP2E1/NOS2/CYP4F2/CYP2C9/HBE1/CYP4Z1/CYP3A4/CYP4Z2P/CYP4A11 | 13 |
| GO:0046906 | tetrapyrrole binding | 13/298 | 145/17697 | 1.069209e-06 | 7.010954e-05 | 6.045451e-05 | CYP4F22/CYP27A1/CYP2C18/CYP4F12/CYP2E1/NOS2/CYP4F2/CYP2C9/HBE1/CYP4Z1/CYP3A4/CYP4Z2P/CYP4A11 | 13 |
| GO:0016705 | oxidoreductase activity, acting on paired donors, with incorporation or reduction of molecular oxygen | 13/298 | 159/17697 | 3.020049e-06 | 1.732753e-04 | 1.494129e-04 | CYP4F22/CYP27A1/CYP2C18/CYP4F12/CYP2E1/NOS2/CYP4F2/CYP2C9/CYP4Z1/CYP3A4/CYP4Z2P/TYR/CYP4A11 | 13 |
| GO:0016712 | oxidoreductase activity, acting on paired donors, with incorporation or reduction of molecular oxygen, reduced flavin or flavoprotein as one donor, and incorporation of one atom of oxygen | 6/298 | 32/17697 | 1.358691e-05 | 6.929325e-04 | 5.975063e-04 | CYP2C18/CYP4F12/CYP2E1/CYP2C9/CYP4Z1/CYP3A4 | 6 |
| GO:0016709 | oxidoreductase activity, acting on paired donors, with incorporation or reduction of molecular oxygen, NAD(P)H as one donor, and incorporation of one atom of oxygen | 6/298 | 39/17697 | 4.430801e-05 | 2.033738e-03 | 1.753664e-03 | CYP27A1/CYP4F12/CYP2E1/NOS2/CYP4F2/CYP4A11 | 6 |
| GO:0005216 | ion channel activity | 18/298 | 416/17697 | 2.581647e-04 | 1.027156e-02 | 8.857026e-03 | CLCA4/SCNN1B/SCNN1G/CACNA1S/HTR3A/BEST2/GABRB2/KCNC1/KCNA2/KCNH5/GRIA2/HTR3B/NMUR2/OTOP1/HCN1/P2RX3/GABRG1/SLC24A5 | 18 |
| GO:0030414 | peptidase inhibitor activity | 11/298 | 182/17697 | 2.685375e-04 | 1.027156e-02 | 8.857026e-03 | SPINK5/SPINK7/SERPINB11/FETUB/PI16/SERPINB12/CRB2/SPINK9/SERPINA4/WFDC10B/SPINK8 | 11 |
| GO:0004745 | retinol dehydrogenase activity | 4/298 | 20/17697 | 3.085350e-04 | 1.089366e-02 | 9.393453e-03 | RDH12/ADH1B/DHRS7C/ADH4 | 4 |
| GO:0061134 | peptidase regulator activity | 12/298 | 219/17697 | 3.523502e-04 | 1.111630e-02 | 9.585432e-03 | SPINK5/MAL/SPINK7/SERPINB11/FETUB/PI16/SERPINB12/CRB2/SPINK9/SERPINA4/WFDC10B/SPINK8 | 12 |
| GO:0022838 | substrate-specific channel activity | 18/298 | 428/17697 | 3.632777e-04 | 1.111630e-02 | 9.585432e-03 | CLCA4/SCNN1B/SCNN1G/CACNA1S/HTR3A/BEST2/GABRB2/KCNC1/KCNA2/KCNH5/GRIA2/HTR3B/NMUR2/OTOP1/HCN1/P2RX3/GABRG1/SLC24A5 | 18 |
| GO:0008395 | steroid hydroxylase activity | 5/298 | 38/17697 | 4.167104e-04 | 1.195438e-02 | 1.030810e-02 | CYP27A1/CYP2C18/CYP2E1/CYP2C9/CYP3A4 | 5 |
| GO:0015276 | ligand-gated ion channel activity | 9/298 | 138/17697 | 5.552793e-04 | 1.374973e-02 | 1.185620e-02 | SCNN1B/SCNN1G/HTR3A/GABRB2/GRIA2/HTR3B/HCN1/P2RX3/GABRG1 | 9 |
| GO:0022834 | ligand-gated channel activity | 9/298 | 138/17697 | 5.552793e-04 | 1.374973e-02 | 1.185620e-02 | SCNN1B/SCNN1G/HTR3A/GABRB2/GRIA2/HTR3B/HCN1/P2RX3/GABRG1 | 9 |
| GO:0022839 | ion gated channel activity | 15/298 | 334/17697 | 5.691608e-04 | 1.374973e-02 | 1.185620e-02 | CLCA4/SCNN1B/SCNN1G/CACNA1S/HTR3A/GABRB2/KCNC1/KCNA2/KCNH5/GRIA2/HTR3B/NMUR2/HCN1/P2RX3/GABRG1 | 15 |
| GO:0022836 | gated channel activity | 15/298 | 343/17697 | 7.474915e-04 | 1.631174e-02 | 1.406539e-02 | CLCA4/SCNN1B/SCNN1G/CACNA1S/HTR3A/GABRB2/KCNC1/KCNA2/KCNH5/GRIA2/HTR3B/NMUR2/HCN1/P2RX3/GABRG1 | 15 |
| GO:0015267 | channel activity | 18/298 | 456/17697 | 7.624274e-04 | 1.631174e-02 | 1.406539e-02 | CLCA4/SCNN1B/SCNN1G/CACNA1S/HTR3A/BEST2/GABRB2/KCNC1/KCNA2/KCNH5/GRIA2/HTR3B/NMUR2/OTOP1/HCN1/P2RX3/GABRG1/SLC24A5 | 18 |
| GO:0022803 | passive transmembrane transporter activity | 18/298 | 457/17697 | 7.818264e-04 | 1.631174e-02 | 1.406539e-02 | CLCA4/SCNN1B/SCNN1G/CACNA1S/HTR3A/BEST2/GABRB2/KCNC1/KCNA2/KCNH5/GRIA2/HTR3B/NMUR2/OTOP1/HCN1/P2RX3/GABRG1/SLC24A5 | 18 |
| GO:0032036 | myosin heavy chain binding | 3/298 | 12/17697 | 9.290484e-04 | 1.854057e-02 | 1.598729e-02 | MYL2/MYL3/AMPD1 | 3 |
| GO:0008307 | structural constituent of muscle | 5/298 | 46/17697 | 1.019812e-03 | 1.926927e-02 | 1.661563e-02 | NEB/MYL2/TCAP/MYOT/MYL3 | 5 |
| GO:0004867 | serine-type endopeptidase inhibitor activity | 7/298 | 94/17697 | 1.049525e-03 | 1.926927e-02 | 1.661563e-02 | SPINK5/SPINK7/SERPINB11/SERPINB12/SPINK9/SERPINA4/SPINK8 | 7 |
| GO:0070330 | aromatase activity | 3/298 | 14/17697 | 1.499243e-03 | 2.646741e-02 | 2.282249e-02 | CYP2C18/CYP4F12/CYP4Z1 | 3 |
| GO:0005230 | extracellular ligand-gated ion channel activity | 6/298 | 75/17697 | 1.648958e-03 | 2.803229e-02 | 2.417187e-02 | HTR3A/GABRB2/GRIA2/HTR3B/P2RX3/GABRG1 | 6 |
| GO:0099094 | ligand-gated cation channel activity | 7/298 | 105/17697 | 1.996002e-03 | 3.272018e-02 | 2.821416e-02 | SCNN1B/SCNN1G/HTR3A/GRIA2/HTR3B/HCN1/P2RX3 | 7 |
| GO:0047498 | calcium-dependent phospholipase A2 activity | 3/298 | 16/17697 | 2.249738e-03 | 3.560793e-02 | 3.070423e-02 | PLA2G4D/PLA2G3/PLA2G2E | 3 |
| GO:0004866 | endopeptidase inhibitor activity | 9/298 | 175/17697 | 2.913230e-03 | 4.334942e-02 | 3.737962e-02 | SPINK5/SPINK7/SERPINB11/FETUB/SERPINB12/CRB2/SPINK9/SERPINA4/SPINK8 | 9 |
| GO:0019825 | oxygen binding | 4/298 | 36/17697 | 3.036327e-03 | 4.334942e-02 | 3.737962e-02 | CYP2C18/CYP2E1/HBE1/CYP3A4 | 4 |
| GO:0005261 | cation channel activity | 13/298 | 319/17697 | 3.061706e-03 | 4.334942e-02 | 3.737962e-02 | SCNN1B/SCNN1G/CACNA1S/HTR3A/KCNC1/KCNA2/KCNH5/GRIA2/HTR3B/OTOP1/HCN1/P2RX3/SLC24A5 | 13 |
| GO:0016755 | transferase activity, transferring amino-acyl groups | 3/298 | 18/17697 | 3.197611e-03 | 4.334942e-02 | 3.737962e-02 | TGM3/TGM6/TGM7 | 3 |
| GO:0008528 | G protein-coupled peptide receptor activity | 8/298 | 146/17697 | 3.345043e-03 | 4.334942e-02 | 3.737962e-02 | CXCR2/CCR4/XCR1/NTSR1/NPY6R/RXFP1/CCKAR/NMUR2 | 8 |
| GO:0051393 | alpha-actinin binding | 4/298 | 37/17697 | 3.359896e-03 | 4.334942e-02 | 3.737962e-02 | NRAP/XIRP2/MYOT/LDB3 | 4 |
| GO:0048018 | receptor ligand activity | 17/298 | 482/17697 | 3.450251e-03 | 4.334942e-02 | 3.737962e-02 | SLURP1/DEFB4A/NTS/ENDOU/CCL19/IL36A/FAM3B/CCL26/SCGB3A1/VGF/GDF10/FGF19/IFNK/FGF6/TTR/UCN3/INSL4 | 17 |
| GO:0022824 | transmitter-gated ion channel activity | 5/298 | 61/17697 | 3.607054e-03 | 4.334942e-02 | 3.737962e-02 | HTR3A/GABRB2/GRIA2/HTR3B/GABRG1 | 5 |
| GO:0022835 | transmitter-gated channel activity | 5/298 | 61/17697 | 3.607054e-03 | 4.334942e-02 | 3.737962e-02 | HTR3A/GABRB2/GRIA2/HTR3B/GABRG1 | 5 |
| GO:0008236 | serine-type peptidase activity | 9/298 | 182/17697 | 3.777727e-03 | 4.334942e-02 | 3.737962e-02 | KLK7/KLK13/PRSS27/ENDOU/TMPRSS11B/TMPRSS2/F7/PCSK2/PLG | 9 |
| GO:0061135 | endopeptidase regulator activity | 9/298 | 182/17697 | 3.777727e-03 | 4.334942e-02 | 3.737962e-02 | SPINK5/SPINK7/SERPINB11/FETUB/SERPINB12/CRB2/SPINK9/SERPINA4/SPINK8 | 9 |
| GO:0001653 | peptide receptor activity | 8/298 | 152/17697 | 4.269827e-03 | 4.759495e-02 | 4.104048e-02 | CXCR2/CCR4/XCR1/NTSR1/NPY6R/RXFP1/CCKAR/NMUR2 | 8 |
| GO:0017171 | serine hydrolase activity | 9/298 | 186/17697 | 4.355094e-03 | 4.759495e-02 | 4.104048e-02 | KLK7/KLK13/PRSS27/ENDOU/TMPRSS11B/TMPRSS2/F7/PCSK2/PLG | 9 |
| GO:0004252 | serine-type endopeptidase activity | 8/298 | 160/17697 | 5.795270e-03 | 6.186114e-02 | 5.334202e-02 | KLK7/KLK13/PRSS27/TMPRSS11B/TMPRSS2/F7/PCSK2/PLG | 8 |
| GO:0005212 | structural constituent of eye lens | 3/298 | 23/17697 | 6.522490e-03 | 6.652939e-02 | 5.736739e-02 | HSPB6/LIM2/CRYGC | 3 |
| GO:0016493 | C-C chemokine receptor activity | 3/298 | 23/17697 | 6.522490e-03 | 6.652939e-02 | 5.736739e-02 | CXCR2/CCR4/XCR1 | 3 |
| GO:0019957 | C-C chemokine binding | 3/298 | 24/17697 | 7.362599e-03 | 7.205071e-02 | 6.212835e-02 | CXCR2/CCR4/XCR1 | 3 |
| GO:0042805 | actinin binding | 4/298 | 46/17697 | 7.377741e-03 | 7.205071e-02 | 6.212835e-02 | NRAP/XIRP2/MYOT/LDB3 | 4 |
| GO:0102567 | phospholipase A2 activity (consuming 1,2-dipalmitoylphosphatidylcholine) | 3/298 | 25/17697 | 8.263783e-03 | 7.740973e-02 | 6.674936e-02 | PLA2G4D/PLA2G3/PLA2G2E | 3 |
| GO:0102568 | phospholipase A2 activity consuming 1,2-dioleoylphosphatidylethanolamine) | 3/298 | 25/17697 | 8.263783e-03 | 7.740973e-02 | 6.674936e-02 | PLA2G4D/PLA2G3/PLA2G2E | 3 |
| GO:0005254 | chloride channel activity | 5/298 | 75/17697 | 8.655770e-03 | 7.945997e-02 | 6.851725e-02 | CLCA4/BEST2/GABRB2/NMUR2/GABRG1 | 5 |
| GO:0001637 | G protein-coupled chemoattractant receptor activity | 3/298 | 26/17697 | 9.226980e-03 | 8.144585e-02 | 7.022965e-02 | CXCR2/CCR4/XCR1 | 3 |
| GO:0004950 | chemokine receptor activity | 3/298 | 26/17697 | 9.226980e-03 | 8.144585e-02 | 7.022965e-02 | CXCR2/CCR4/XCR1 | 3 |
| GO:0003785 | actin monomer binding | 3/298 | 28/17697 | 1.134265e-02 | 9.823164e-02 | 8.470381e-02 | MYL2/LMOD2/MYL3 | 3 |
| GO:0015280 | ligand-gated sodium channel activity | 2/298 | 10/17697 | 1.163149e-02 | 9.886770e-02 | 8.525228e-02 | SCNN1B/SCNN1G | 2 |
