# Supplementary material for: DDX59-AS1 is a prognostic biomarker and correlated with immune infiltrates in OSCC
Source: Front Genet. 2022 Aug 23;13:892727. doi: 10.3389/fgene.2022.892727 (PMC9447487; doi:10.3389/fgene.2022.892727)
Supplement: Supplementary file 8 [file Table3.docx]

| ID | Description | GeneRatio | BgRatio | pvalue | p.adjust | qvalue | geneID | Count |
| --- | --- | --- | --- | --- | --- | --- | --- | --- |
| GO:0031424 | keratinization | 35/310 | 224/18670 | 4.909051e-24 | 1.302371e-20 | 1.230880e-20 | KRT13/KRT1/KRT10/SPRR3/KRT4/DSG1/TGM3/SPINK5/KLK13/KRT78/RPTN/FLG/LCE3A/KRT2/KRT3/LCE1F/LCE1C/LCE2B/KRT76/LCE2C/LCE2A/LCE1A/HRNR/LCE2D/LCE1B/LCE6A/KRT36/LCE1E/KRT72/SPINK9/KRTAP5-4/KRTAP3-2/KRTAP9-4/LCE3B/KRTAP9-8 | 35 |
| GO:0030216 | keratinocyte differentiation | 37/310 | 305/18670 | 2.002479e-21 | 2.656288e-18 | 2.510476e-18 | KRT13/KRT1/KRT10/SPRR3/KRT4/DSG1/TGM3/SPINK5/KLK13/KRT78/RPTN/FLG/LCE3A/KRT2/KRT3/C1orf68/LCE1F/ACER1/LCE1C/LCE2B/KRT76/LCE2C/LCE2A/LCE1A/HRNR/LCE2D/LCE1B/LCE6A/KRT36/LCE1E/KRT72/SPINK9/KRTAP5-4/KRTAP3-2/KRTAP9-4/LCE3B/KRTAP9-8 | 37 |
| GO:0018149 | peptide cross-linking | 20/310 | 60/18670 | 3.161997e-21 | 2.796260e-18 | 2.642764e-18 | KRT1/KRT10/SPRR3/TGM3/FLG/LCE3A/KRT2/C1orf68/LCE1F/LCE1C/LCE2B/LCE2C/LCE2A/LCE1A/LCE2D/LCE1B/TGM6/TGM7/LCE1E/LCE3B | 20 |
| GO:0043588 | skin development | 42/310 | 419/18670 | 5.376272e-21 | 3.565812e-18 | 3.370074e-18 | KRT13/KRT1/KRT10/SPRR3/KRT4/DSG1/TGM3/SPINK5/KLK13/KRT78/ALOX12B/RPTN/FLG/ALOX12/LCE3A/KRT2/FLG2/KRT3/C1orf68/LCE1F/ACER1/LCE1C/LCE2B/KRT76/LCE2C/LCE2A/LCE1A/HRNR/ALX4/LCE2D/LCE1B/LCE6A/KRT36/LCE1E/KRT72/SPINK9/ASCL4/KRTAP5-4/KRTAP3-2/KRTAP9-4/LCE3B/KRTAP9-8 | 42 |
| GO:0008544 | epidermis development | 42/310 | 464/18670 | 2.524429e-19 | 1.339462e-16 | 1.265935e-16 | KRT13/KRT1/KRT10/SPRR3/KRT4/DSG1/TGM3/SPINK5/CALML5/KLK7/KLK13/KRT78/RPTN/FLG/LCE3A/KRT2/FLG2/KRT3/C1orf68/LCE1F/ACER1/LCE1C/LCE2B/KRT76/LCE2C/LCE2A/LCE1A/HRNR/ALX4/LCE2D/LCE1B/LCE6A/KRT36/LCE1E/DCT/KRT72/SPINK9/KRTAP5-4/KRTAP3-2/KRTAP9-4/LCE3B/KRTAP9-8 | 42 |
| GO:0009913 | epidermal cell differentiation | 37/310 | 358/18670 | 4.932627e-19 | 2.181043e-16 | 2.061319e-16 | KRT13/KRT1/KRT10/SPRR3/KRT4/DSG1/TGM3/SPINK5/KLK13/KRT78/RPTN/FLG/LCE3A/KRT2/KRT3/C1orf68/LCE1F/ACER1/LCE1C/LCE2B/KRT76/LCE2C/LCE2A/LCE1A/HRNR/LCE2D/LCE1B/LCE6A/KRT36/LCE1E/KRT72/SPINK9/KRTAP5-4/KRTAP3-2/KRTAP9-4/LCE3B/KRTAP9-8 | 37 |
| GO:0070268 | cornification | 18/310 | 112/18670 | 3.904078e-13 | 1.479646e-10 | 1.398423e-10 | KRT13/KRT1/KRT10/SPRR3/KRT4/DSG1/SPINK5/KLK13/KRT78/RPTN/FLG/KRT2/KRT3/KRT76/LCE1A/KRT36/KRT72/SPINK9 | 18 |
| GO:0050891 | multicellular organismal water homeostasis | 13/310 | 68/18670 | 7.851148e-11 | 2.603637e-08 | 2.460715e-08 | KRT1/ALOX12B/FLG/SCNN1B/ALOX12/FLG2/SCNN1G/CYP4F12/ACER1/BPIFA1/HRNR/CYP4F2/CYP4A11 | 13 |
| GO:0030104 | water homeostasis | 13/310 | 74/18670 | 2.395889e-10 | 7.062548e-08 | 6.674862e-08 | KRT1/ALOX12B/FLG/SCNN1B/ALOX12/FLG2/SCNN1G/CYP4F12/ACER1/BPIFA1/HRNR/CYP4F2/CYP4A11 | 13 |
| GO:0033561 | regulation of water loss via skin | 7/310 | 26/18670 | 1.631594e-07 | 4.328618e-05 | 4.091006e-05 | KRT1/ALOX12B/FLG/ALOX12/FLG2/ACER1/HRNR | 7 |
| GO:0001676 | long-chain fatty acid metabolic process | 12/310 | 109/18670 | 2.643862e-07 | 6.376514e-05 | 6.026486e-05 | ALOX12B/ALOX12/HPGD/CYP2C18/CYP4F12/CYP2E1/SLC27A6/ACSL6/CYP4F2/CYP2C9/CYP3A4/CYP4A11 | 12 |
| GO:0019373 | epoxygenase P450 pathway | 6/310 | 20/18670 | 6.361424e-07 | 1.406405e-04 | 1.329203e-04 | CYP2C18/CYP4F12/CYP2E1/CYP4F2/CYP2C9/CYP4A11 | 6 |
| GO:0061436 | establishment of skin barrier | 6/310 | 23/18670 | 1.588607e-06 | 3.241979e-04 | 3.064017e-04 | KRT1/ALOX12B/FLG/ALOX12/FLG2/HRNR | 6 |
| GO:0019369 | arachidonic acid metabolic process | 8/310 | 51/18670 | 1.807383e-06 | 3.424991e-04 | 3.236982e-04 | ALOX12B/ALOX12/CYP2C18/CYP4F12/CYP2E1/CYP4F2/CYP2C9/CYP4A11 | 8 |
| GO:0006690 | icosanoid metabolic process | 11/310 | 114/18670 | 3.122114e-06 | 5.521979e-04 | 5.218860e-04 | ALOX12B/CYP4F22/PLA2G3/ALOX12/HPGD/CYP2C18/CYP4F12/CYP2E1/CYP4F2/CYP2C9/CYP4A11 | 11 |
| GO:0042759 | long-chain fatty acid biosynthetic process | 6/310 | 30/18670 | 8.472736e-06 | 1.340412e-03 | 1.266832e-03 | ALOX12B/ALOX12/HPGD/CYP2E1/CYP2C9/CYP3A4 | 6 |
| GO:0032309 | icosanoid secretion | 7/310 | 45/18670 | 8.589144e-06 | 1.340412e-03 | 1.266832e-03 | PLA2G3/NTSR1/NOS2/CYP4F2/NMUR2/CYP4A11/PLA2G2E | 7 |
| GO:0071715 | icosanoid transport | 7/310 | 49/18670 | 1.536107e-05 | 2.144890e-03 | 2.027150e-03 | PLA2G3/NTSR1/NOS2/CYP4F2/NMUR2/CYP4A11/PLA2G2E | 7 |
| GO:1901571 | fatty acid derivative transport | 7/310 | 49/18670 | 1.536107e-05 | 2.144890e-03 | 2.027150e-03 | PLA2G3/NTSR1/NOS2/CYP4F2/NMUR2/CYP4A11/PLA2G2E | 7 |
| GO:1901568 | fatty acid derivative metabolic process | 12/310 | 167/18670 | 2.350064e-05 | 3.117359e-03 | 2.946238e-03 | ALOX12B/CYP4F22/PLA2G3/ALOX12/HPGD/CYP2C18/CYP4F12/CYP2E1/ACSL6/CYP4F2/CYP2C9/CYP4A11 | 12 |
| GO:0046717 | acid secretion | 10/310 | 124/18670 | 4.234453e-05 | 5.349525e-03 | 5.055873e-03 | PLA2G3/NTSR1/NOS2/SLC9A4/CYP4F2/SLC1A7/AGXT/NMUR2/CYP4A11/PLA2G2E | 10 |
| GO:0031640 | killing of cells of other organism | 7/310 | 62/18670 | 7.314731e-05 | 8.437383e-03 | 7.974228e-03 | DEFB4A/LCE3A/ARG1/MUC7/NOS2/HTN3/LCE3B | 7 |
| GO:0044364 | disruption of cells of other organism | 7/310 | 62/18670 | 7.314731e-05 | 8.437383e-03 | 7.974228e-03 | DEFB4A/LCE3A/ARG1/MUC7/NOS2/HTN3/LCE3B | 7 |
| GO:0033559 | unsaturated fatty acid metabolic process | 9/310 | 110/18670 | 9.139766e-05 | 1.010325e-02 | 9.548650e-03 | ALOX12B/ALOX12/HPGD/CYP2C18/CYP4F12/CYP2E1/CYP4F2/CYP2C9/CYP4A11 | 9 |
| GO:0019372 | lipoxygenase pathway | 4/310 | 16/18670 | 1.158881e-04 | 1.193954e-02 | 1.128414e-02 | ALOX12B/PLA2G3/ALOX12/HPGD | 4 |
| GO:0006936 | muscle contraction | 17/310 | 360/18670 | 1.170102e-04 | 1.193954e-02 | 1.128414e-02 | NEB/MYH7/MYL2/TCAP/HSPB6/TRDN/ATP2A1/MYOT/ATP1A2/LMOD2/CACNA1S/ADRA2A/MYL3/STRIT1/NMUR2/P2RX3/GHSR | 17 |
| GO:0015908 | fatty acid transport | 8/310 | 97/18670 | 2.120458e-04 | 2.025928e-02 | 1.914719e-02 | PLA2G3/NTSR1/NOS2/SLC27A6/CYP4F2/NMUR2/CYP4A11/PLA2G2E | 8 |
| GO:0055078 | sodium ion homeostasis | 6/310 | 52/18670 | 2.138183e-04 | 2.025928e-02 | 1.914719e-02 | SCNN1B/ATP1A2/SCNN1G/CYP4F12/CYP4F2/CYP4A11 | 6 |
| GO:0015837 | amine transport | 8/310 | 102/18670 | 2.995854e-04 | 2.694870e-02 | 2.546940e-02 | RHCG/ATP1A2/ADRA2A/ARG1/NTSR1/SLC38A3/KCNA2/GHSR | 8 |
| GO:0003012 | muscle system process | 19/310 | 465/18670 | 3.047347e-04 | 2.694870e-02 | 2.546940e-02 | NEB/MYH7/MYL2/TCAP/HSPB6/TRDN/ATP2A1/MYOT/ATP1A2/LMOD2/CACNA1S/ADRA2A/MYL3/PI16/STRIT1/MYOC/NMUR2/P2RX3/GHSR | 19 |
| GO:0015718 | monocarboxylic acid transport | 10/310 | 162/18670 | 3.859891e-04 | 3.303320e-02 | 3.121990e-02 | PLA2G3/NTSR1/NOS2/SLC38A3/SLC27A6/CYP4F2/SLC6A13/NMUR2/CYP4A11/PLA2G2E | 10 |
| GO:0032890 | regulation of organic acid transport | 6/310 | 59/18670 | 4.294292e-04 | 3.455416e-02 | 3.265737e-02 | ATP1A2/ARG1/NTSR1/SLC38A3/CYP4F2/CYP4A11 | 6 |
| GO:0030049 | muscle filament sliding | 5/310 | 39/18670 | 4.428350e-04 | 3.455416e-02 | 3.265737e-02 | NEB/MYH7/MYL2/TCAP/MYL3 | 5 |
| GO:0033275 | actin-myosin filament sliding | 5/310 | 39/18670 | 4.428350e-04 | 3.455416e-02 | 3.265737e-02 | NEB/MYH7/MYL2/TCAP/MYL3 | 5 |
| GO:0015849 | organic acid transport | 15/310 | 333/18670 | 4.807360e-04 | 3.542757e-02 | 3.348284e-02 | ATP1A2/PLA2G3/ARG1/FOLR3/NTSR1/NOS2/SLC38A3/SLC27A6/CYP4F2/SLC1A7/SLC6A13/AGXT/NMUR2/CYP4A11/PLA2G2E | 15 |
| GO:0046942 | carboxylic acid transport | 15/310 | 333/18670 | 4.807360e-04 | 3.542757e-02 | 3.348284e-02 | ATP1A2/PLA2G3/ARG1/FOLR3/NTSR1/NOS2/SLC38A3/SLC27A6/CYP4F2/SLC1A7/SLC6A13/AGXT/NMUR2/CYP4A11/PLA2G2E | 15 |
| GO:0042758 | long-chain fatty acid catabolic process | 3/310 | 10/18670 | 4.989400e-04 | 3.570068e-02 | 3.374096e-02 | CYP4F12/CYP4F2/CYP4A11 | 3 |
| GO:0048871 | multicellular organismal homeostasis | 19/310 | 485/18670 | 5.113555e-04 | 3.570068e-02 | 3.374096e-02 | KRT1/ALOX12B/FLG/RDH12/SCNN1B/ALOX12/PLAC8/FLG2/SCNN1G/ZG16B/CYP4F12/ACER1/NTSR1/BPIFA1/HRNR/CYP4F2/SFTPA2/AIPL1/CYP4A11 | 19 |
| GO:0042572 | retinol metabolic process | 5/310 | 41/18670 | 5.609947e-04 | 3.816202e-02 | 3.606719e-02 | RDH12/ADH1B/CYP3A4/ADH4/TTR | 5 |
| GO:0006937 | regulation of muscle contraction | 10/310 | 171/18670 | 5.906653e-04 | 3.917587e-02 | 3.702539e-02 | MYH7/MYL2/HSPB6/ATP2A1/ATP1A2/ADRA2A/MYL3/STRIT1/NMUR2/GHSR | 10 |
| GO:0042738 | exogenous drug catabolic process | 4/310 | 24/18670 | 6.093181e-04 | 3.942734e-02 | 3.726305e-02 | CYP2C18/CYP2E1/CYP2C9/CYP3A4 | 4 |
| GO:0046185 | aldehyde catabolic process | 3/310 | 11/18670 | 6.776276e-04 | 4.180805e-02 | 3.951307e-02 | AGXT/ADH4/AGXT2 | 3 |
| GO:1901569 | fatty acid derivative catabolic process | 3/310 | 11/18670 | 6.776276e-04 | 4.180805e-02 | 3.951307e-02 | CYP4F12/CYP4F2/CYP4A11 | 3 |
| GO:0070296 | sarcoplasmic reticulum calcium ion transport | 5/310 | 43/18670 | 7.013650e-04 | 4.228912e-02 | 3.996774e-02 | TRDN/ATP2A1/ATP1A2/STRIT1/DHRS7C | 5 |
| GO:0055003 | cardiac myofibril assembly | 4/310 | 26/18670 | 8.351668e-04 | 4.923772e-02 | 4.653491e-02 | NEB/MYL2/TCAP/NRAP | 4 |
| GO:0006721 | terpenoid metabolic process | 8/310 | 120/18670 | 8.882127e-04 | 5.122670e-02 | 4.841471e-02 | RDH12/CYP2C18/CYP2E1/ADH1B/CYP2C9/CYP3A4/ADH4/TTR | 8 |
| GO:0002026 | regulation of the force of heart contraction | 4/310 | 27/18670 | 9.677045e-04 | 5.462383e-02 | 5.162535e-02 | MYH7/MYL2/ATP1A2/MYL3 | 4 |
| GO:0042391 | regulation of membrane potential | 17/310 | 434/18670 | 9.990970e-04 | 5.522092e-02 | 5.218967e-02 | TRDN/ATP1A2/ALOX12/NTSR1/HTR3A/BEST2/CLDN19/GABRB2/KCNC1/KCNA2/KCNH5/HTR3B/MYOC/HCN1/UCN3/P2RX3/GABRG1 | 17 |
| GO:0051952 | regulation of amine transport | 7/310 | 95/18670 | 1.032110e-03 | 5.588141e-02 | 5.281390e-02 | ATP1A2/ADRA2A/ARG1/NTSR1/SLC38A3/KCNA2/GHSR | 7 |
| GO:0030239 | myofibril assembly | 6/310 | 73/18670 | 1.336850e-03 | 7.093327e-02 | 6.703952e-02 | NEB/MYL2/TCAP/NRAP/LDB3/LMOD2 | 6 |
| GO:1901616 | organic hydroxy compound catabolic process | 6/310 | 74/18670 | 1.434875e-03 | 7.350029e-02 | 6.946563e-02 | CYP27A1/CYP4F12/NTSR1/CYP4F2/ADH4/CYP4A11 | 6 |
| GO:0014819 | regulation of skeletal muscle contraction | 3/310 | 14/18670 | 1.440639e-03 | 7.350029e-02 | 6.946563e-02 | MYH7/ATP2A1/STRIT1 | 3 |
| GO:0006691 | leukotriene metabolic process | 4/310 | 31/18670 | 1.646870e-03 | 7.943903e-02 | 7.507837e-02 | ALOX12/CYP4F12/CYP4F2/CYP4A11 | 4 |
| GO:0050482 | arachidonic acid secretion | 4/310 | 31/18670 | 1.646870e-03 | 7.943903e-02 | 7.507837e-02 | PLA2G3/NTSR1/NMUR2/PLA2G2E | 4 |
| GO:1903963 | arachidonate transport | 4/310 | 31/18670 | 1.646870e-03 | 7.943903e-02 | 7.507837e-02 | PLA2G3/NTSR1/NMUR2/PLA2G2E | 4 |
| GO:0006631 | fatty acid metabolic process | 15/310 | 383/18670 | 1.953807e-03 | 9.256161e-02 | 8.748061e-02 | ALOX12B/PLA2G4D/PLA2G3/ALOX12/HPGD/CYP2C18/CYP4F12/CYP2E1/SLC27A6/ACSL6/CYP4F2/CYP2C9/CYP3A4/CYP4A11/GHSR | 15 |
| GO:0051955 | regulation of amino acid transport | 4/310 | 33/18670 | 2.086745e-03 | 9.712518e-02 | 9.179367e-02 | ATP1A2/ARG1/NTSR1/SLC38A3 | 4 |
| GO:0001977 | renal system process involved in regulation of blood volume | 3/310 | 16/18670 | 2.162528e-03 | 9.724043e-02 | 9.190260e-02 | CYP4F12/CYP4F2/CYP4A11 | 3 |
| GO:0032305 | positive regulation of icosanoid secretion | 3/310 | 16/18670 | 2.162528e-03 | 9.724043e-02 | 9.190260e-02 | NTSR1/CYP4F2/CYP4A11 | 3 |
| GO:0043270 | positive regulation of ion transport | 12/310 | 275/18670 | 2.254332e-03 | 9.882418e-02 | 9.339941e-02 | TRDN/ATP2A1/ADRA2A/NTSR1/HTR3A/STRIT1/SLC38A3/CYP4F2/KCNC1/PIRT/CYP4A11/P2RX3 | 12 |
| GO:0006720 | isoprenoid metabolic process | 8/310 | 139/18670 | 2.272248e-03 | 9.882418e-02 | 9.339941e-02 | RDH12/CYP2C18/CYP2E1/ADH1B/CYP2C9/CYP3A4/ADH4/TTR | 8 |
| GO:0032892 | positive regulation of organic acid transport | 4/310 | 34/18670 | 2.334442e-03 | 9.989151e-02 | 9.440815e-02 | NTSR1/SLC38A3/CYP4F2/CYP4A11 | 4 |
