# Supplementary material for: DDX59-AS1 is a prognostic biomarker and correlated with immune infiltrates in OSCC
Source: Front Genet. 2022 Aug 23;13:892727. doi: 10.3389/fgene.2022.892727 (PMC9447487; doi:10.3389/fgene.2022.892727)
Supplement: Supplementary file 9 [file Table4.docx]

| ID | Description | GeneRatio | BgRatio | pvalue | p.adjust | qvalue | geneID | Count |
| --- | --- | --- | --- | --- | --- | --- | --- | --- |
| GO:0001533 | cornified envelope | 21/329 | 65/19717 | 7.705958e-22 | 2.489024e-19 | 2.230672e-19 | KRT1/KRT10/SPRR3/DSG1/RPTN/FLG/LCE3A/KRT2/FLG2/C1orf68/LCE1F/LCE1C/LCE2B/LCE2C/LCE2A/LCE1A/HRNR/LCE2D/LCE1B/LCE1E/LCE3B | 21 |
| GO:0045095 | keratin filament | 12/329 | 95/19717 | 6.039302e-08 | 6.668420e-06 | 5.976260e-06 | KRT13/KRT1/KRT4/KRT78/KRT2/KRT3/KRT76/KRT72/KRTAP5-4/KRTAP3-2/KRTAP9-4/KRTAP9-8 | 12 |
| GO:0030017 | sarcomere | 17/329 | 204/19717 | 6.193579e-08 | 6.668420e-06 | 5.976260e-06 | NEB/MYH7/MYL2/CMYA5/TCAP/NRAP/XIRP2/MYOT/LDB3/LMOD2/CACNA1S/C10orf71/KLHL40/MYL3/MYOZ3/SMTNL1/ABRA | 17 |
| GO:0005882 | intermediate filament | 17/329 | 214/19717 | 1.242197e-07 | 1.003074e-05 | 8.989586e-06 | KRT13/KRT1/KRT10/KRT4/KRT78/FLG/KRT2/KRT3/KRT76/NEFH/GFAP/KRT36/KRT72/KRTAP5-4/KRTAP3-2/KRTAP9-4/KRTAP9-8 | 17 |
| GO:0044449 | contractile fiber part | 17/329 | 221/19717 | 1.973747e-07 | 1.275041e-05 | 1.142696e-05 | NEB/MYH7/MYL2/CMYA5/TCAP/NRAP/XIRP2/MYOT/LDB3/LMOD2/CACNA1S/C10orf71/KLHL40/MYL3/MYOZ3/SMTNL1/ABRA | 17 |
| GO:0030016 | myofibril | 17/329 | 224/19717 | 2.393332e-07 | 1.288410e-05 | 1.154678e-05 | NEB/MYH7/MYL2/CMYA5/TCAP/NRAP/XIRP2/MYOT/LDB3/LMOD2/CACNA1S/C10orf71/KLHL40/MYL3/MYOZ3/SMTNL1/ABRA | 17 |
| GO:0043292 | contractile fiber | 17/329 | 234/19717 | 4.445920e-07 | 2.051474e-05 | 1.838538e-05 | NEB/MYH7/MYL2/CMYA5/TCAP/NRAP/XIRP2/MYOT/LDB3/LMOD2/CACNA1S/C10orf71/KLHL40/MYL3/MYOZ3/SMTNL1/ABRA | 17 |
| GO:0031674 | I band | 13/329 | 143/19717 | 8.394204e-07 | 3.389160e-05 | 3.037376e-05 | NEB/MYH7/TCAP/NRAP/XIRP2/MYOT/LDB3/CACNA1S/C10orf71/KLHL40/MYL3/MYOZ3/SMTNL1 | 13 |
| GO:0045111 | intermediate filament cytoskeleton | 17/329 | 251/19717 | 1.182005e-06 | 4.242085e-05 | 3.801771e-05 | KRT13/KRT1/KRT10/KRT4/KRT78/FLG/KRT2/KRT3/KRT76/NEFH/GFAP/KRT36/KRT72/KRTAP5-4/KRTAP3-2/KRTAP9-4/KRTAP9-8 | 17 |
| GO:0031672 | A band | 6/329 | 39/19717 | 4.229042e-05 | 1.365980e-03 | 1.224196e-03 | MYL2/CMYA5/LMOD2/KLHL40/MYL3/SMTNL1 | 6 |
| GO:0016324 | apical plasma membrane | 15/329 | 318/19717 | 3.144914e-04 | 9.234612e-03 | 8.276090e-03 | DSG1/RHCG/CEACAM6/CEACAM5/MAL/CLCA4/SCNN1B/UPK1B/SCNN1G/CEACAM7/CYP4F12/CRB2/SLC9A4/CYP4F2/CYP4A11 | 15 |
| GO:0030018 | Z disc | 9/329 | 132/19717 | 3.782562e-04 | 9.517825e-03 | 8.529906e-03 | NEB/MYH7/TCAP/NRAP/XIRP2/MYOT/LDB3/C10orf71/MYOZ3 | 9 |
| GO:1902495 | transmembrane transporter complex | 15/329 | 324/19717 | 3.830703e-04 | 9.517825e-03 | 8.529906e-03 | ATP2A1/SCNN1B/ATP1A2/SCNN1G/CACNA1S/HTR3A/BEST2/GABRB2/KCNC1/KCNA2/SHISA9/GRIA2/HTR3B/HCN1/GABRG1 | 15 |
| GO:0033017 | sarcoplasmic reticulum membrane | 5/329 | 39/19717 | 4.534836e-04 | 1.046251e-02 | 9.376540e-03 | TRDN/ATP2A1/STRIT1/DHRS7C/ART1 | 5 |
| GO:1990351 | transporter complex | 15/329 | 332/19717 | 4.942068e-04 | 1.064192e-02 | 9.537324e-03 | ATP2A1/SCNN1B/ATP1A2/SCNN1G/CACNA1S/HTR3A/BEST2/GABRB2/KCNC1/KCNA2/SHISA9/GRIA2/HTR3B/HCN1/GABRG1 | 15 |
| GO:0034702 | ion channel complex | 14/329 | 301/19717 | 5.685779e-04 | 1.118280e-02 | 1.002207e-02 | ATP2A1/SCNN1B/SCNN1G/CACNA1S/HTR3A/BEST2/GABRB2/KCNC1/KCNA2/SHISA9/GRIA2/HTR3B/HCN1/GABRG1 | 14 |
| GO:0031225 | anchored component of membrane | 10/329 | 170/19717 | 5.885687e-04 | 1.118280e-02 | 1.002207e-02 | CEACAM6/CEACAM5/PRSS27/LYPD2/CEACAM7/FOLR3/CNTN5/TREH/ART1/GP2 | 10 |
| GO:0045177 | apical part of cell | 16/329 | 384/19717 | 7.776739e-04 | 1.395493e-02 | 1.250645e-02 | DSG1/RHCG/CEACAM6/CEACAM5/MAL/VCAM1/CLCA4/SCNN1B/UPK1B/SCNN1G/CEACAM7/CYP4F12/CRB2/SLC9A4/CYP4F2/CYP4A11 | 16 |
| GO:0043679 | axon terminus | 8/329 | 119/19717 | 8.704692e-04 | 1.479798e-02 | 1.326200e-02 | CALB1/NTS/TNN/NTSR1/KCNC1/KCNA2/UCN3/P2RX3 | 8 |
| GO:0016529 | sarcoplasmic reticulum | 6/329 | 71/19717 | 1.187750e-03 | 1.879193e-02 | 1.684139e-02 | CMYA5/TRDN/ATP2A1/STRIT1/DHRS7C/ART1 | 6 |
| GO:0034703 | cation channel complex | 11/329 | 220/19717 | 1.221767e-03 | 1.879193e-02 | 1.684139e-02 | ATP2A1/SCNN1B/SCNN1G/CACNA1S/HTR3A/KCNC1/KCNA2/SHISA9/GRIA2/HTR3B/HCN1 | 11 |
| GO:0016528 | sarcoplasm | 6/329 | 80/19717 | 2.199721e-03 | 3.152534e-02 | 2.825311e-02 | CMYA5/TRDN/ATP2A1/STRIT1/DHRS7C/ART1 | 6 |
| GO:0044306 | neuron projection terminus | 8/329 | 138/19717 | 2.244838e-03 | 3.152534e-02 | 2.825311e-02 | CALB1/NTS/TNN/NTSR1/KCNC1/KCNA2/UCN3/P2RX3 | 8 |
| GO:0042599 | lamellar body | 3/329 | 17/19717 | 2.631425e-03 | 3.541460e-02 | 3.173868e-02 | SPINK5/KLK7/SFTPA2 | 3 |
| GO:0099240 | intrinsic component of synaptic membrane | 8/329 | 164/19717 | 6.380473e-03 | 8.243571e-02 | 7.387916e-02 | HTR3A/CNTN5/GABRB2/KCNC1/KCNA2/SHISA9/P2RX3/GHSR | 8 |
| GO:0044305 | calyx of Held | 3/329 | 24/19717 | 7.185933e-03 | 8.927139e-02 | 8.000532e-02 | CALB1/KCNC1/KCNA2 | 3 |
| GO:0042383 | sarcolemma | 7/329 | 136/19717 | 7.901828e-03 | 9.452927e-02 | 8.471745e-02 | VCAM1/MYOT/ATP1A2/ALOX12/CACNA1S/SLC27A6/FGF6 | 7 |
| GO:0062023 | collagen-containing extracellular matrix | 14/329 | 406/19717 | 8.609073e-03 | 9.931181e-02 | 8.900358e-02 | KRT1/FLG/TNXB/CILP/MMRN1/COL4A4/HMCN2/SERPINB12/HRNR/COL6A5/GDF10/F7/MYOC/PLG | 14 |
