# Supplementary material for: DDX59-AS1 is a prognostic biomarker and correlated with immune infiltrates in OSCC
Source: Front Genet. 2022 Aug 23;13:892727. doi: 10.3389/fgene.2022.892727 (PMC9447487; doi:10.3389/fgene.2022.892727)
Supplement: Supplementary file 10 [file Table6.docx]

| ID | Description | GeneRatio | BgRatio | pvalue | p.adjust | qvalue | geneID | Count |
| --- | --- | --- | --- | --- | --- | --- | --- | --- |
| hsa00590 | Arachidonic acid metabolism | 9/129 | 63/8075 | 5.851840e-07 | 0.0001170368 | 0.0001028692 | ALOX12B/PLA2G4D/PLA2G3/ALOX12/CYP2E1/CYP4F2/CYP2C9/CYP4A11/PLA2G2E | 9 |
| hsa00591 | Linoleic acid metabolism | 6/129 | 29/8075 | 5.198155e-06 | 0.0005198155 | 0.0004568905 | PLA2G4D/PLA2G3/CYP2E1/CYP2C9/CYP3A4/PLA2G2E | 6 |
| hsa00830 | Retinol metabolism | 8/129 | 68/8075 | 1.128837e-05 | 0.0007525578 | 0.0006614587 | RDH12/CYP2C18/ADH1B/CYP2C9/CYP3A4/ADH4/CYP4A11/UGT2B28 | 8 |
| hsa04970 | Salivary secretion | 9/129 | 93/8075 | 1.588750e-05 | 0.0007943749 | 0.0006982137 | CALML5/ATP1A2/MUC5B/DMBT1/MUC7/BEST2/PRH2/HTN3/AMY2A | 9 |
| hsa04726 | Serotonergic synapse | 9/129 | 115/8075 | 8.644877e-05 | 0.0034579509 | 0.0030393569 | ALOX12B/PLA2G4D/ALOX12/CYP2C18/CACNA1S/HTR3A/CYP2C9/GABRB2/HTR3B | 9 |
| hsa04972 | Pancreatic secretion | 8/129 | 102/8075 | 2.127446e-04 | 0.0069693528 | 0.0061256943 | CLCA4/ATP2A1/ATP1A2/PLA2G3/CCKAR/CPA2/AMY2A/PLA2G2E | 8 |
| hsa00500 | Starch and sucrose metabolism | 5/129 | 36/8075 | 2.439273e-04 | 0.0069693528 | 0.0061256943 | PYGM/GYS2/TREH/G6PC/AMY2A | 5 |
| hsa05204 | Chemical carcinogenesis | 7/129 | 82/8075 | 3.172398e-04 | 0.0079309941 | 0.0069709264 | CYP2C18/CYP2E1/ADH1B/CYP2C9/CYP3A4/ADH4/UGT2B28 | 7 |
| hsa04260 | Cardiac muscle contraction | 7/129 | 87/8075 | 4.564859e-04 | 0.0101441322 | 0.0089161583 | MYH7/MYL2/TRDN/ATP2A1/ATP1A2/CACNA1S/MYL3 | 7 |
| hsa05150 | Staphylococcus aureus infection | 7/129 | 96/8075 | 8.270285e-04 | 0.0164756804 | 0.0144812559 | KRT13/KRT10/DSG1/DEFB4A/DEFB4B/KRT36/PLG | 7 |
| hsa00982 | Drug metabolism - cytochrome P450 | 6/129 | 71/8075 | 9.061624e-04 | 0.0164756804 | 0.0144812559 | CYP2E1/ADH1B/CYP2C9/CYP3A4/ADH4/UGT2B28 | 6 |
| hsa04080 | Neuroactive ligand-receptor interaction | 14/129 | 341/8075 | 1.045919e-03 | 0.0174319784 | 0.0153217916 | SLURP1/NTS/ADRA2A/NTSR1/RXFP1/CCKAR/GABRB2/GRIA2/NMUR2/PLG/UCN3/P2RX3/GABRG1/GHSR | 14 |
| hsa00980 | Metabolism of xenobiotics by cytochrome P450 | 6/129 | 77/8075 | 1.387752e-03 | 0.0213500259 | 0.0187655491 | CYP2E1/ADH1B/CYP2C9/CYP3A4/ADH4/UGT2B28 | 6 |
| hsa00350 | Tyrosine metabolism | 4/129 | 36/8075 | 2.464866e-03 | 0.0352123737 | 0.0309498232 | ADH1B/DCT/ADH4/TYR | 4 |
| hsa00071 | Fatty acid degradation | 4/129 | 44/8075 | 5.149474e-03 | 0.0686596544 | 0.0603482226 | ADH1B/ACSL6/ADH4/CYP4A11 | 4 |
| hsa00592 | alpha-Linolenic acid metabolism | 3/129 | 25/8075 | 7.085819e-03 | 0.0885727327 | 0.0778507703 | PLA2G4D/PLA2G3/PLA2G2E | 3 |
